# Targeting multiple signaling pathways: the new approach to acute myeloid leukemia therapy

**DOI:** 10.1038/s41392-020-00361-x

**Published:** 2020-12-18

**Authors:** Jenna L. Carter, Katie Hege, Jay Yang, Hasini A. Kalpage, Yongwei Su, Holly Edwards, Maik Hüttemann, Jeffrey W. Taub, Yubin Ge

**Affiliations:** 1 Cancer Biology Graduate Program, Wayne State University School of Medicine, Detroit, MI USA; 2 MD/PhD Program, Wayne State University School of Medicine, Detroit, MI USA; 3 Department of Oncology, Wayne State University School of Medicine, Detroit, MI USA; 4 Molecular Therapeutics Program, Barbara Ann Karmanos Cancer Institute, Wayne State University School of Medicine, Detroit, MI USA; 5 Center for Molecular Medicine and Genetics, Wayne State University School of Medicine, Detroit, MI USA; 6 National Engineering Laboratory for AIDS Vaccine, Key Laboratory for Molecular Enzymology and Engineering, The Ministry of Education, School of Life Sciences, Jilin University, Changchun, China; 7 Division of Pediatric Hematology/Oncology, Children’s Hospital of Michigan, Detroit, MI USA; 8 Department of Pediatrics, Wayne State University School of Medicine, Detroit, MI USA

**Keywords:** Haematological cancer, Cancer therapy

## Abstract

Acute myeloid leukemia (AML) is the most common form of acute leukemia in adults and the second most common form of acute leukemia in children. Despite this, very little improvement in survival rates has been achieved over the past few decades. This is partially due to the heterogeneity of AML and the need for more targeted therapeutics than the traditional cytotoxic chemotherapies that have been a mainstay in therapy for the past 50 years. In the past 20 years, research has been diversifying the approach to treating AML by investigating molecular pathways uniquely relevant to AML cell proliferation and survival. Here we review the development of novel therapeutics in targeting apoptosis, receptor tyrosine kinase (RTK) signaling, hedgehog (HH) pathway, mitochondrial function, DNA repair, and c-Myc signaling. There has been an impressive effort into better understanding the diversity of AML cell characteristics and here we highlight important preclinical studies that have supported therapeutic development and continue to promote new ways to target AML cells. In addition, we describe clinical investigations that have led to FDA approval of new targeted AML therapies and ongoing clinical trials of novel therapies targeting AML survival pathways. We also describe the complexity of targeting leukemia stem cells (LSCs) as an approach to addressing relapse and remission in AML and targetable pathways that are unique to LSC survival. This comprehensive review details what we currently understand about the signaling pathways that support AML cell survival and the exceptional ways in which we disrupt them.

## Introduction

### Epidemiology of acute myeloid leukemia (AML)

There are about 20,000 new cases of AML diagnosed each year in the United States.^1,2^ AML can affect people of all ages, but it is much more common in older adults with the age-adjusted incidence for those aged ≥65 years being 20.1 per 100,000 person-years compared with 2.0 per 100,000 person-years for those aged <65 years. The median age at diagnosis is 68 years and is most frequently diagnosed among people aged 65–74 years. Additionally, incidence is modestly increased in males compared with females and in Caucasians compared with other ethnic groups.^3^

In most patients, the precise inciting event(s) leading to AML is unknown, but a genetic origin is strongly implicated. Environmental factors including exposure to chemicals such as benzene have also been associated with AML. Patients with a prior history of myelodysplastic syndromes (MDS) or myeloproliferative neoplasms (MPN) and those who have previously received radiation and/or chemotherapy are also at increased risk of developing AML. Collectively, patients with AML with an antecedent myeloid disorder and those with therapy-related AML are considered to have secondary AML. In large population-based studies, 25% of AML cases are considered secondary and these are associated with lower rates of response to therapy and an inferior prognosis compared with de novo AML.^4^ AML can also be seen in patients with inherited genetic syndromes such as Fanconi’s anemia, Bloom syndrome, Down syndrome, and others.^5–7^ There has also been an increased interest in the study of inherited predispositions to myeloid malignancies such as those seen with familial mutations of *CEBPA*, *DDX4*, and *RUNX1*.^8^

Premalignant evidence of clonal hematopoiesis can be found in healthy individuals, as evidenced by acquired mutations in genes such as *DNMT3A*, *TET2*, and *ASXL1*.^9^ The prevalence of such clonal hematopoiesis of indeterminate potential (CHIP) increases with age and is associated with an increased risk of developing a subsequent hematological malignancy, particularly MDS or AML. However, the large majority of people who have CHIP never develop a hematologic cancer.^10^

### AML pathobiology

AML results from the malignant transformation of myeloid precursor cells that are driven by a number of acquired genetic abnormalities. The biology is complex and involves a number of interdependent genetic mechanisms and pathways. The two-hit model of leukemogenesis hypothesizes that many cases of AML are the result of the cooperation of two types of mutations: mutations that result in unhindered cell proliferation (class I mutations such as FMS-like tyrosine kinase 3 (*FLT3*), *NRAS*, *c-KIT*) and mutations that result in the arrest of normal myeloid differentiation (class II mutations such as *RUNX1-RUNX1T1*, *CEBPA*, *TP53*).^11^ Although this two-hit model is a simplification of the biology of AML, it serves as a useful conceptual framework.

Cytogenetic abnormalities can be found in 50–60% of AML cases and strongly correlate with prognosis.^12^ For example, the core-binding factor (CBF) leukemias with the balanced translocations t(8;21)(q22;q22), inv(16)(p13;q22), and t(16;16)(p13;q22) are associated with a favorable prognosis while AML with deletion 7, a monosomal karyotype, or a complex karyotype are associated with a dismal prognosis.^13^ The overwhelming majority of AML cases are also typified by genetic mutations such as *NPM1*, *FLT3*, *isocitrate dehydrogenase 1* (*IDH1*), *IDH2* and *TP53*.^14^ Together these acquired cytogenetic and molecular abnormalities encode transcription factors, tumor suppressors, DNA repair proteins, signaling molecules, regulators of apoptosis, and other diverse mechanisms, which promote leukemia development.

AML appears to be maintained by a pool of self-renewing leukemia stem cells (LSCs) that are typically characterized by a CD34+CD38−CD123+ immunophenotype.^15^ AML stem cells are highly resistant to chemotherapy since they are primarily in the G0 phase of the cell cycle and preferentially express multiple drug-resistant proteins such as P-glycoprotein and Bcl-2.^16,17^ The persistence of these LSCs predisposes to relapse even if bulk AML cells are effectively eliminated by treatment. As discussed below, there is great interest in therapies targeting LSCs in order to prevent relapse.

Patients with AML usually present with manifestations of cytopenias due to bone marrow (BM) failure such as fatigue, fevers, infection, or bleeding. AML can sometimes present with signs and symptoms of hyperleukocytosis, which usually affects the pulmonary and central nervous systems. Extramedullary collections of leukemic blasts (e.g., myeloid sarcoma) is much less common but can occur in almost any organ, with skin (aka. leukemia cutis) and lymph node involvement being the most common.^18^ The diagnosis of AML is typically made by documenting ≥20% myeloblasts in a BM biopsy or aspirate specimen using adjunctive tests such as flow cytometry, cytogenetics, and fluorescence in situ hybridization to confirm and categorize the leukemia.^18^ Molecular profiling of AML is now considered mandatory in the era of precision medicine. There are a number of platforms available but next-generation sequencing panels focused on the most critical and common gene mutations are now routinely being employed. At a minimum, the European LeukemiaNet recommends testing for *FLT3-ITD*, *FLT3-TKD*, *TP53*, *NPM1*, *RUNX1*, *ASXL1*, and *CEBPA* mutations.^19^ One could also argue that screening for *IDH1* and *IDH2* mutations should be considered essential particularly at the time of relapse due to the availability of IDH1 and IDH2 inhibitors.

### AML classification

The original FAB (French–American–British) classification of AML was the first attempt to systematically categorize this disease and divided AML into groups (FAB M0–M7) largely based on morphology and a few histochemical stains. The modern World Health Organization (WHO) classification is based on a combination of morphology, immunophenotype, clinical characteristics, and genetics with the goal of identifying distinct biologic entities of AML with defined molecular pathways.^20^ The WHO classification recognizes six major categories of AML: (a) AML with recurrent genetic abnormalities; (b) AML with myelodysplasia-related features; (c) therapy-related AML and MDS; (d) AML, not otherwise specified; (e) myeloid sarcoma; and (f) myeloid proliferations related to Down syndrome.

There are currently 11 genetic subtypes of AML recognized in the WHO classification including t(8;21)(q22;q22), inv(16)(p13;q22), t(16;16)(p13;q22), and several others. AML with the following gene mutations have also been included: *NPM1*, *CEBPA* (biallelic), *BCR-ABL1*, and *RUNX1*. AML with *NPM1* or biallelic *CEBPA* mutations are considered favorable while AML with *RUNX1* mutations are unfavorable.^21,22^

Although AML with *FLT3* mutation is not included in the WHO classification as a distinct entity, it is the most commonly (~30% of AML) mutated gene in AML and its presence predicts an unfavorable prognosis.^23^ *FLT3* internal tandem duplication (*FLT3-ITD)* mutations result in a constitutively active FLT3, a transmembrane tyrosine kinase, which in turns results in the growth and proliferation of leukemia cells.^24^ Because of its association with high rates of relapse, allogeneic hematopoietic stem cell transplant (SCT) is generally recommended in first remission. *FLT3-ITD* mutations are also an example of the complex interplay of genetic abnormalities seen in AML and their diverse effects on outcomes. Many of these mutations are often found in the same patient. *NPM1* mutations can often co-exist with *FLT3-ITD* mutations resulting in a genotype with an intermediate-risk prognosis, depending on the *FLT3* allelic ratio.^25^

About 5–10% of AML patients have acute promyelocytic leukemia (APL) with *PML-RARA* fusion gene. This is characterized by a reciprocal translocation between chromosomes 15 and 17 (t(15;17)(q24;q21)) resulting in the production of a *PML-RARA* fusion gene. APL remains the paradigm of the genetic classification and treatment of AML given its disease-defining molecular signature and excellent outcomes with targeted therapies. APL is clinically characterized by disseminated intravascular coagulation and hyperfibrinolysis, which can result in a potentially fatal hemorrhagic diathesis. However, if managed promptly and appropriately, the majority of patients are cured with treatment regimens that include a combination of targeted biologic therapies including all-trans retinoic acid and arsenic trioxide.^26^ Due to the unique characteristics of APL with *PML-RARA* fusion gene, this entity is not specifically covered in the remainder of this review.

### Treatment of AML

**Fig. 1:**
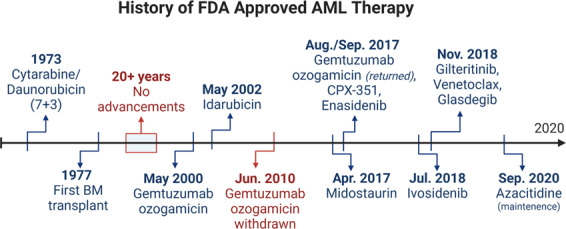
History of AML therapies. Timeline of approved clinical therapies in the United States for the treatment of AML

The standard treatment for newly diagnosed AML remained static for many decades and was divided into induction therapy and consolidation therapy (Fig. 1). The goals of induction therapy are achievement of a complete morphologic remission, which results in the restoration of normal hematopoiesis and allows for subsequent therapy that maximizes the probability of long-term remission and potentially a cure.

A combination of a daunorubicin and cytarabine was introduced approximately half a century ago and remained the standard therapy for most patients until very recently (Fig. 1). The most common iteration of this combination consists of 7 days of infusional cytarabine and 3 days of daunorubicin, the so-called “7+3” regimen. Remission rates are reported between 30 and 80% depending on patient and disease-related factors but long-term survivals and cure rates are appreciably lower due to relapses. This intensive chemotherapy approach is accompanied by a number of potential complications, including prolonged marrow aplasia, profound cytopenias, need for transfusional support, and risks of neutropenic infection and sepsis. Mortality rates during induction range from 5% reported in clinical trials of younger patients to >20% reported in analyses of real-world data from the SEER database.^27,28^

In patients who achieve a complete remission (CR), the cure rate approaches zero in the absence of some form of post-remission therapy. The antimetabolite cytarabine has been the mainstay of consolidation therapy for decades. A higher dose of cytarabine such as HIDAC improves survival in younger patients and in those who carry favorable cytogenetics.^29^ SCT is often used as post-remission therapy since its application is associated with the lowest rates of leukemia relapse. This benefit is balanced by risks of transplant-related mortality, including age, pre-existing comorbidities, and other molecular lesions, and by risks of long-term complications in the form of graft-vs-disease. Retrospective analyses have clearly shown a survival benefit in favor of SCT in patients with AML who have adverse-risk genetic abnormalities (intermediate- or poor-risk AML).^30^ The absence of randomized data makes definitive conclusions in the intermediate-risk patient population less than clear-cut; however, most experts would favor SCT in eligible patients in the absence of contraindications.

Older patients (>65 years) with AML have a much poorer prognosis compared with their younger counterparts primarily due to an increased prevalence of adverse-risk genetic features that promote resistance to chemotherapy.^31^ Intensive chemotherapy in this population is associated with lower rates of remission, increased toxicities, higher rates of early mortality, and dismal long-term survival rates in the absence of transplantation.^31,32^ For this reason, less intensive therapies such as single-agent hypomethylating agents (HMAs) and low-dose cytarabine (LDAC) have been used prior to the development and recent approval of novel agents.

HMAs (azacitidine and decitabine) are commonly used in the United States for the first-line treatment of AML. However, their use as a single-agent therapy for AML has always been met with a lack of enthusiasm given the modest activity of these drugs. HMAs are associated with a CR rate of approximately 17.8 and 27.8% for decitabine and azacitidine, respectively, a duration of remission of 10.4 months (azacitidine), and an overall survival (OS) of 7.7 and 10.4 months for decitabine and azacitidine, respectively, compared to no responses in supportive care patients.^33,34^ Overall results with LDAC are likely to be worse, though it has improved OS and higher rates of supportive care in elderly adults who cannot receive conventional therapy.^35,36^ However patients with adverse cytogenetics have no benefit in remission or survival on LDAC.^36^ Single-agent HMA use has not brought clear improvements in survival in AML patients and limited Food and Drug Administration (FDA) approval for this use.^33,34^ Fortunately, novel drugs such as venetoclax, an inhibitor of the antiapoptotic protein Bcl-2, appear to markedly potentiate the activity of HMA or LDAC making venetoclax-based combinations a more attractive option for older patients.^37,38^ HMAs are continued to be used in combination therapies and will be highlighted extensively in this review.

Unlike acute lymphoblastic leukemia (ALL), there has been no role for maintenance therapy in AML. However, maintenance with an oral form of azacitidine, CC-486, was recently reported to improve survival compared with placebo in patients who completed standard 7+3 induction and cytarabine consolidation.^39^ This therapy was just recently (September 2020) approved for maintenance therapy in adults with AML in first remission and is likely to be considered standard of care for these patients (Fig. 1). In the subset of patients with *FLT3* mutations, tyrosine kinase inhibitors (TKIs) are often used as posttransplantation maintenance despite the lack of regulatory approval. Additionally, there are several clinical trials investigating new potential AML maintenance therapies (NCT01546038, NCT03564821, NCT03881735, NCT03515512, NCT03728335, NCT03932643).

### AML clinical outcomes

The OS of all AML patients is around 30% but it is quite dependent on age: long-term survivals for patients less than and older than 65 years is about 50% and 10%, respectively.^40^ Survival rates in children are about 60–65%. Age acts as a surrogate for a panoply of patient and disease-related factors.^41^ Patient variables include performance status, comorbidities, and impaired organ function. Disease-related factors include clinical characteristics and biological features, such as cytogenetics and genetic mutations. Patients can be divided into adverse-, intermediate-, and favorable-risk groups based on cytogenetics and molecular features; however, much heterogeneity exists even within these subgroups.^19^ There has been a consistent and substantial improvement in the survival of younger patients over the past several decades despite the lack of drug approvals during this time period.^40^ The improvement is likely due to better supportive care measures and implementation and improvement of the risk-adapted use of transplantation.

Significant improvements in the elderly AML population have been much harder to come by. The adverse-risk biology of AML in older adults is associated with increased drug resistance leading to early mortality, shorter remissions, and inferior survivals. This has led to a therapeutic nihilism in some treating physicians when faced with the prospect of treating older patients with either intensive chemotherapy, which is deemed too toxic, or less intensive therapies with less than desired effectiveness. Fortunately, recent drug approvals have improved the treatment landscape of AML, particularly in older patients, so that almost all newly diagnosed patients should be offered some form of therapy.

### Newly approved AML therapies

For many years, treatment options for AML remained stagnant, with the standard 7+3 regimen of cytarabine and daunorubicin and SCT being the only option for patients (Fig. 1). The difficulty in developing new therapies for AML attributes mainly to myelosuppression, which probably is the biggest hurdle overall in developing new drugs for this deadly disease. This is due to a significant amount of overlap in processes and signaling between AML cells and normal hematopoietic cells.^42^ Fortunately, the past few years have seen the development of more targeted therapies to better address the pathobiology and heterogeneity of AML and includes the FDA approval of midostaurin, gemtuzumab ozogamicin, CPX-351, enasidenib, ivosidenib, gilteritinib, venetoclax, and glasdegib (Fig. 1). This surge in approved AML therapies has diversified the treatment options for patients and marks a turning point in how AML is approached.

#### FLT3 inhibitors: midostaurin and gilteritinib

As mentioned, *FLT3* mutations are common in AML, occurring in approximately 30% of patients, and can be due to ITD mutations or point mutations in the tyrosine kinase domain (TKD).^43^ Constitutive activation of the mutant FLT3 supports tumorigenesis in hematopoietic precursor cells, so inhibition of FLT3 has been heavily pursued as a targeted therapeutic option for these patients.^43^ Midostaurin and gilteritinib are type I FLT3 inhibitors and are effective against FLT3-ITD and FLT3-TKD.^44^ Midostaurin was approved by the FDA for therapy in adult patients with newly diagnosed *FLT3*-mutated AML in April 2017 and was followed by the approval of gilteritinib for the treatment of adult patients with relapsed/refractory (R/R) AML with *FLT3* mutations in November 2018 (Fig. 1).^45,46^

#### IDH1 and IDH2 inhibitors: enasidenib and ivosidenib

Mutations in *IDH1* and *IDH2* occur in about 20% of AML patients and are common in other malignancies, such as glioblastoma.^47^ These mutant enzymes have become promising targets for new therapies, as they promote tumorigenesis in cells through production of the oncometabolite, 2-hydroxyglutarate (2-HG).^48^ Inhibition of the mutant IDH1 and IDH2 enzymes is able to reduce the production of 2-HG to normal physiologic levels, and this promotes the differentiation of leukemia cells.^48^ Enasidenib (AG-221), an IDH2 mutant inhibitor, and ivosidenib (AG-120), an IDH1 mutant inhibitor, were approved for treatment of R/R AML in August 2017 and July 2018, respectively (Fig. 1).^49,50^

#### Bcl-2 inhibitor: venetoclax

AML has been shown to be dependent on dysregulations of the apoptotic pathway, including overexpression of Bcl-2, which is an important antiapoptotic protein.^51^ This has supported the development of Bcl-2 inhibitors to promote the induction of apoptosis in AML cells and has led to the discovery of venetoclax, a potent and selective Bcl-2 inhibitor.^52,53^ After being approved for use in chronic lymphocytic leukemia (CLL) (2016), demonstrating promising results in early clinical trials, and being well tolerated in older patients, venetoclax was FDA approved in November 2018 for the treatment of AML (Fig. 1).^37,54,55^ It was approved for use in combination with azacitidine or decitabine or LDAC for the treatment of newly diagnosed AML patients aged ≥75 years or who have comorbidities that preclude the use of intensive induction chemotherapy. Currently, venetoclax is being studied in numerous clinical trials in combination and single-agent therapies.

#### Hedgehog (HH) pathway inhibition: glasdegib

Aberrant activation of the HH signaling pathway has been shown to be increased in AML cells and has also been correlated with worse prognosis and drug resistance in AML.^56,57^ Numerous studies have demonstrated that targeting this pathway showed antitumor activity and combination with current therapies improves efficacy.^56^ This led to the development of glasdegib, a HH pathway inhibitor that works by binding to and inhibiting the transmembrane protein Smoothened (SMO).^58^ Glasdegib was FDA approved in November 2018 for use in combination with LDAC for the treatment of newly diagnosed AML patients aged ≥75 years or who have comorbidities that preclude the use of intensive induction chemotherapy (Fig. 1).^59^

#### Antibody–drug conjugate (ADC): gemtuzumab ozogamicin

CD33 has been found to be highly expressed on the leukemia cells of most AML patients and has developed as a targetable antigen.^60^ Gemtuzumab ozogamicin is an ADC of a CD33-directed humanized monoclonal antibody (mAb) that is covalently linked to *N*-acetyl gamma calicheamicin (cytotoxic drug). The antibody portion localizes to CD33 antigens found on myeloid leukemia blasts and calicheamicin is internalized and induces double-strand breaks in DNA and cell death. Gemtuzumab ozogamicin was originally approved for monotherapy in CD33+ AML patients aged ≥60 years in May 2000 but was withdrawn from the market in 2010 due to concerns about toxicity. Additional studies using gemtuzumab ozogamicin at lower doses in combination with currently approved therapies confirmed its safety. This led to the approval of gemtuzumab ozogamicin in September 2017 for the treatment of CD33-positive AML in adults and pediatric patients aged ≥2 years (Fig. 1).^61^

#### Cytotoxic therapy: CPX-351

CPX-351 is a liposomal formulation of cytarabine and daunorubicin, two standard of care chemotherapy drugs used in the treatment of AML. CPX-351 utilizes liposomal-encapsulated delivery system to avoid the first-pass metabolism, enhancing the pharmacodynamics and pharmacokinetics (PK) of the drugs and potentially leading to greater efficacy.^62^ Given its success in clinical trials in improving patient response rate and survival, CPX-351 was FDA approved in August 2017 for the treatment of adults with newly diagnosed AML with myelodysplasia-related changes or therapy-related AML (Fig. 1).^62,63^

## Targeting signaling pathways in AML

### Apoptotic pathways

**Fig. 2:**
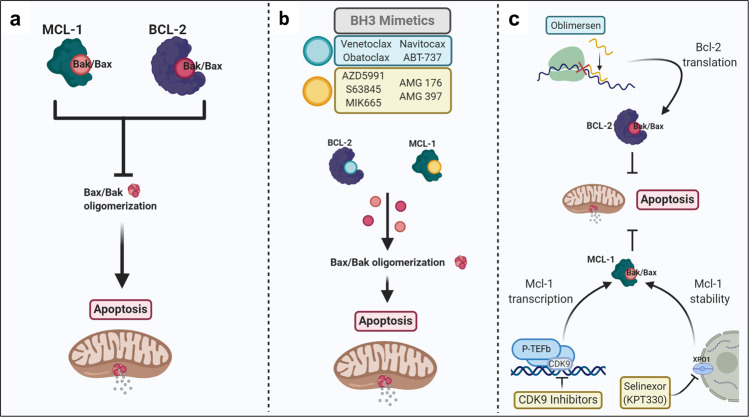
Targeting antiapoptotic proteins induces apoptosis in AML. **a** Antiapoptotic proteins Mcl-1 and Bcl-2 bind and sequester apoptotic effector proteins Bak/Bax to prevent Bak/Bax oligomerization and subsequent induction of apoptosis. **b** BH3 mimetics bind to the BH3-binding site of antiapoptotic proteins, Bcl-2 and Mcl-1, and release Bax/Bak to promote oligomerization and MOMP, which leads to subsequent induction of apoptosis. **c** Oblimersen is an anti-sense oligonucleotide that binds specifically to *Bcl-2* mRNA to prevent Bcl-2 translation and promote AML cell apoptosis. Selinexor (KPT330) inhibits XPO1 expression and subsequently decreases Mcl-1 stability to promote AML cell apoptosis. CDK9 inhibitors prevent the transcription of *Mcl-1* gene to promote apoptosis of AML cells

Apoptosis is essential for ensuring the homeostasis of healthy tissue via two highly regulated pathways—the mitochondrial (intrinsic) pathway and death receptor (DR; extrinsic) pathway. The extrinsic pathway of apoptosis is initiated by DRs or tumor necrosis factor (TNF) family receptors, which are cell surface receptors that are activated by ligand interactions. Activation of these receptors by external stimuli results in the recruitment and activation of caspase 8 and ultimately leads to cell death. The intrinsic pathway is usually initiated in a cell-autonomous way when a cell undergoes stress and is unable to repair damages. The intrinsic apoptosis pathway is governed by the Bcl-2 family proteins, which consists of highly regulated proapoptotic and antiapoptotic proteins. Proapoptotic effectors Bak and Bax are necessary to initiate apoptosis in the cell and, when activated, will form pores on the outer membrane of mitochondria. This process is referred to as mitochondrial outer membrane permeabilization and results in release of cytochrome *c*, a proapoptotic factor, from the mitochondrial intermembrane space into the cytosol. Cytochrome *c* promotes assembly of the apoptosome and activation of caspase 9—ultimately leading to cell death. Antiapoptotic proteins (e.g., Bcl-2, Bcl-xL, and myeloid cell leukemia 1 (Mcl-1)) bind, sequester, and inhibit proapoptotic proteins (Bak/Bax) to prevent apoptosis. BH3-only proteins (e.g., Bid, Bim, Bad, and Noxa) enhance apoptotic activity through activation of proapoptotic effectors or neutralization of antiapoptotic proteins. The imbalance of interactions between these proapoptotic and antiapoptotic proteins control the caspase cascade and cell death (Fig. 2a).^64^

Apoptosis is frequently dysregulated in cancer. Cancer cells evade apoptosis through different mechanisms, but many involve overexpression of antiapoptotic proteins (Bcl-2, Mcl-1, and Bcl-xL) or loss of expression of proapoptotic proteins (Bak/Bax).^65^ These dysregulations promote cancer cell survival during treatment as many cancer therapies are dependent on intrinsic apoptosis induction to promote cancer cell death.^51,65,66^ It has been well established in preclinical studies that both Bcl-2 and Mcl-1 are frequently overexpressed in AML cells.^67^ Overexpression of Bcl-2 and/or Mcl-1 is related to poor prognoses and are also associated with chemotherapy resistance. This understanding has led to the development of many therapeutics to target the dysregulated intrinsic apoptotic pathway for the treatment of AML, and here we summarize the targeting of Bcl-2 and Mcl-1.

#### Bcl-2 inhibitors

The antiapoptotic protein Bcl-2 was found to be overexpressed and critical for leukemogenesis of AML.^67^ It is frequently overexpressed in leukemia progenitor and stem cells and in myeloid leukemia blasts in comparison to normal hematopoietic cells.^68^ True to its nature of preventing apoptosis, Bcl-2 has also been shown to play a role in resistance to chemotherapy of AML.^69^ Based on the importance of Bcl-2, many pharmaceutical companies have developed methods to target Bcl-2 for the treatment of AML.

Oblimersen is a single-stranded anti-sense oligonucleotide (ASO) that is complementary to the first six codons of the open reading frame of the *Bcl-2* mRNA sequence. The binding of oblimersen to the *Bcl-2* mRNA targets the duplex for cleavage by RNase H and prevents translation into the Bcl-2 protein (Fig. 2c).^70^ In preclinical studies, oblimersen was shown to decrease *Bcl-2* mRNA to nearly undetectable ranges and showed promising efficacy in in vivo leukemia xenograft models.^70,71^ Following the promising preclinical data, oblimersen was the first drug targeting Bcl-2 to be used for clinical trials in AML. A phase I trial combined oblimersen with standard of care chemotherapy (cytarabine and daunorubicin) and showed that 48% of patients achieved CR, with a median survival of 12.6 months. BM samples from patients who achieved CR had higher expression levels of Bcl-2 prior to induction therapy and showed significant decreases in *Bcl-2* mRNA following treatment. Oblimersen had no additional toxicities compared to the known standard chemotherapy toxicities and no cardiac toxicities were noted.^72^ A phase III randomized trial of intensive induction and consolidation chemotherapy ± oblimersen was performed in 503 untreated older AML patients (>60 years).^73^ The study found that the addition of oblimersen to induction and consolidation therapy resulted in no significant improvement in CR rates, OS, or disease-free survival. These results limited further study of oblimersen in AML, but investigation into Bcl-2 inhibition continued.

With a greater understanding of the intrinsic apoptotic pathway came a new method of targeting Bcl-2 through the development of “BH3 mimetic” small-molecule inhibitors. As mentioned above, BH3 only proteins (BIM, PUMA, BIC, NOXA, BID, etc.) activate apoptosis by binding to the BH3-binding site of antiapoptotic proteins and activating BAX/BAK to oligomerize in mitochondrial outer membrane. The identification of this function led to the development of small molecules that could mimic BH3 only proteins and have been thus termed BH3 mimetics (Fig. 2b).^66^ The effort to develop these small molecules have been immense and has now resulted in seven BH3 mimetics entering clinical trials and the FDA approval of the Bcl-2 inhibitor, venetoclax (Fig. 1).^66^

The first BH3 mimetic tested in clinical trial of AML was obatoclax, which was thought to inhibit Bcl-2 and other antiapoptotic proteins (Bcl-xL, Bcl-w, Bcl-b, A1, and Mcl-1) (Fig. 2b).^74^ In preclinical studies, obatoclax was shown to inhibit cell growth and induce apoptosis in both AML cell lines and primary patient samples^75^ and was able to potentiate the cytotoxic effect of cytarabine in AML cells.^76^ A phase I study of obatoclax included patients with myelodysplasia or refractory leukemia, including AML, ALL, and CLL. This study looked at the efficacy of obatoclax via continuous intravenous (IV) infusion at increasing doses and frequencies.^77^ Obatoclax was well tolerated, but only one AML patient achieved CR and there was no improvement in disease progression. Another phase I/II trial of single agent obatoclax in untreated older patients (≥70 years) with AML was performed to determine a maximum tolerated dose (MTD) and schedule, safety, and efficacy.^78^ Unfortunately, no patients achieved CR in this study and, though obatoclax was tolerable, some neurologic and psychiatric adverse events were observed. Furthermore, additional studies demonstrated that obatoclax can induce apoptosis in the absence of Bax/Bak or Bim, suggesting that obatoclax has off-target effects that contribute to its cytotoxicity.^75,79^

Another potent BH3 mimetic molecule is ABT-737, which was discovered by Abbott Laboratories (now AbbVie) through screening with nuclear magnetic resonance. ABT-737 was shown to bind to Bcl-2, Bcl-xL, and Bcl-w with a high affinity (Fig. 2b).^80^ ABT-737 induces Bak/Bax-dependent apoptosis in AML cells and has activity in vivo.^81^ Unfortunately, due to a lack of oral bioavailability and water insolubility, ABT-737 lacked clinical potential. ABT-263 (navitoclax) is a novel BH3 mimetic with a better oral and bioavailability in comparison to ABT-737.^81^ In a phase I study of patients with R/R CLL, navitoclax was evaluated via dose escalation. The study found that 35% of patients achieved a partial response (PR) and 24% maintained stable disease for at least 6 months.^82^ However, severe thrombocytopenia was observed as a result of Bcl-xL inhibition and was noted to be a major dose-limiting toxicity. Though no clinical trials for ABT-263 in AML have been pursued, authors of the study noted that Bcl-2 remained to be a promising therapeutic target that should be further evaluated.

The greater understanding of subtle differences among the hydrophobic pockets in the antiapoptotic Bcl-2 family proteins led to the discovery and development of the selective Bcl-2 inhibitor venetoclax (also known as ABT-199), manufactured by AbbVie.^53^ Venetoclax was shown to have diverse antitumor activity while sparing platelets. Importantly, venetoclax was shown to induce apoptosis in AML cells in a Bcl-2-dependent mechanism (Fig. 2b).^83^ These preclinical studies demonstrated that venetoclax was an effective orally available therapy against several AML cell lines and primary patient samples, including AML progenitor cells, and in mouse xenograft models. A phase II trial of venetoclax in patients with R/R AML or AML patients who were unfit for intensive chemotherapy demonstrated that venetoclax has clinical activity with acceptable tolerability (NCT01994837).^55^ In this small study (*n* = 32), the overall response rate (ORR) was 19%, with 6% achieving CR and 13% achieving a CR with incomplete hematologic recovery (CRi). Interestingly, 33% of patients with IDH1/2 mutations (4/12) went into CR or CRi. Venetoclax therapy was well tolerated in patients, with some serious adverse events, including febrile neutropenia and hypokalemia. Though there was some response as a monotherapy, 63% of patients experienced treatment failure and the 6-month leukemia-free survival rate was only 10% with a median leukemia-free survival of 2.3 months. The 6-month OS was 36% with a median OS of 4.7 months.^55^

In efforts to combine Bcl-2 inhibition with approved AML therapies, venetoclax was found to synergistically induce apoptosis when combined with cytarabine in AML cell lines and primary patient samples.^84^ In a phase Ib/II clinical trial (NCT02287233), untreated AML patients aged >60 years were enrolled and treated with venetoclax in 28-day cycles and was combined with LDAC subcutaneously on days 1 to 10.^38^ The ORR was 54%, with 26% achieving CR and 28% achieving CRi. The median OS was 10.1 months for all patients and 13.5 months for patients without prior HMA treatment. Consistent with expectations, the common adverse events were hematologic and included febrile neutropenia, thrombocytopenia, neutropenia, and anemia. Previous studies also found evidence that Bcl-2 inhibition sensitized AML cells to HMA ex vivo,^85^ such as azacitidine or decitabine, which has led to clinical trials of these combinations. In a similar non-randomized, open-label, phase Ib study (NCT02203773) of venetoclax containing dose escalation and expansion phases, untreated older patients (age >65 years) ineligible for intensive chemotherapy, with poor-risk cytogenetics in 49% of patients, were enrolled.^37^ During dose escalation, oral venetoclax was administered at 400, 800, or 1200 mg daily in combination with either decitabine or azacitidine. The ORR was 67%, with 37% achieving CR and 30% achieving CRi. Specially, CR+CRi rate was 73% in the 400 mg venetoclax + HMA group. The OR was 60% in patients with poor-risk cytogenetics. Finally, the median OS was 17.5 months, and the median duration of response was 11.3 months. Common adverse events were expected and included febrile neutropenia, hypokalemia, and leukopenia. This study found that venetoclax and HMAs showed promising activity and compared favorably to reported phase III trials of HMA monotherapy and warranted further investigations. Other studies have found similar results when venetoclax was used in combination with LDAC or HMA treatment—supporting combination therapies of venetoclax and current standard of therapies.^86,87^

Based on the phase Ib/II results, a phase III trial of venetoclax plus LDAC compared to LDAC alone was performed (NCT03069352).^88^ This confirmatory trial (VIALE-C) was designed to compare the safety and efficacy of venetoclax or placebo + LDAC in previously untreated patients with AML (≥75 years or adults with comorbidities precluding intensive chemotherapy). Patients with median age of 76 years were recruited, 38% of patients had secondary AML and 20% had prior HMA treatment. In this study, CR and CRi were both 48% for the venetoclax + LDAC arm and 13 and 15%, respectively, for the LDAC alone arm. The median OS for the venetoclax + LDAC arm and LDAC alone were 8.4 and 4.1 months, respectively. Key adverse events (venetoclax + LDAC arm vs LDAC alone) were febrile neutropenia (32% vs 29%), neutropenia (47% vs 16%), and thrombocytopenia (45% vs 37%).^88^ Though these reported results seemed promising, further report by AbbVie announced that this study did not demonstrate statistically significant improvement in OS, with final OS of the study being 7.2 months for venetoclax + LDAC compared to 4.1 months in LDAC alone. While the study results were not significant, they were indicative of clinical activity of venetoclax in combination with LDAC.

A phase III confirmatory trial (VIALE-A) was designed to evaluate the efficacy and safety of azacitidine + venetoclax combination regimen compared to azacitidine and placebo in previously untreated patients with AML who were ineligible for intensive induction therapy (NCT02993523). In this study, the median age was 76 years and median OS in the azacitidine + venetoclax arm was 14.7 months compared to only 9.6 months in the azacitidine alone arm. Incidence of CR/CRi was higher in the combination arm compared to control (66.4% vs 28.3%).^89^ Overall safety of the combination was consistent with the known side effects of each agent and of the population age, and no significant differences were noted in quality of life measures between the two groups though management and monitoring of myelosuppression is important for patient safety with the combination. These findings demonstrated the efficacy of the combination of venetoclax and azacitidine in older patients, though authors highlight the need for future investigations based on varied genomic characteristics, previous treatment with an HMA, and a need for increased patient numbers. Additionally, further studies are needed to determine the effect that venetoclax + HMAs have on long-term survival in elderly AML patients.^89^

In November 2018, the FDA approved venetoclax in combination with LDAC, azacitidine, or decitabine for the treatment of elderly patients with AML who are aged ≥75 years or unfit for intensive chemotherapy. This was an accelerated approval that may be contingent on verification of use in clinical trials, including VIALE-A and VIALE-C. Though there has not been an update to its approval status since these results have been published, the results from these combined trials may modify its accelerated approval and lead to other approvals worldwide.

#### Mcl-1 inhibitors

Mcl-1 is an antiapoptotic member of the Bcl-2 family proteins. Mcl-1 plays an important role in control of apoptosis and has been shown to be essential in early embryology and development and maintenance of both B and T lymphocytes.^90^ It also has been shown to be critical for the survival of leukemic cells, specifically being important in AML. AML cells have been found to be dependent on Mcl-1 for both disease development and persistence, and Mcl-1 is highly expressed in patients with untreated AML.^91,92^ Mcl-1 expression has also been related to cancer cell resistance to therapy and worse prognosis in other hematologic malignancies.^93–95^ These studies have demonstrated that Mcl-1 is a promising therapeutic target in AML and has supported the development of Mcl-1 selective BH3 mimetics and indirect modulators (Fig. 2b, c).

As preclinical studies have shown that Mcl-1 is important in AML survival,^91^ the development of Mcl-1 inhibitors has been a major focus in the field of AML therapy. The first selective Mcl-1 inhibitor, S63845, was discovered in 2016 and showed promising antitumor activity in diverse cancer models.^96^ Since then, other selective Mcl-1 inhibitors including AZD5991, MIK665, AMG 176, and AMG 397, have been discovered (Fig. 2b).^97–100^ Mcl-1 inhibitor screens to determine cancer cell susceptibility have shown that hematological malignancies, such as AML, are sensitive to Mcl-1 inhibition—opening up several phase I studies for Mcl-1 inhibitors.^101,102^ These include AZD5991 (NCT03218683), MIK665/S64315 (NCT02979366, NCT03672695), AMG 176 (NCT02675452, NCT03797261), and AMG 397 (NCT03465540).

AZD5991 is a macrocyclic molecule that directly binds to Mcl-1 with high affinity and is specific to Mcl-1 over other Bcl-2 family proteins.^97^ Upon binding to Mcl-1, AZD5991 promotes the release of Bak from the Mcl-1/Bak complex and rapidly initiates intrinsic apoptosis (Fig. 2b).^97^ Cytotoxicity of AZD5991 was shown to be highest in hematologic malignancies and more specifically in AML. Preclinical studies demonstrated that AZD5991 has a dose-dependent effect in in vivo mouse xenograft models and reduced tumor size by 100% following a single dose (100 mg/kg per IV) and showed efficacy against AML cells in the BM of mice.^97^ A phase I clinical trial is currently ongoing for monotherapy and combinational therapy with venetoclax (NCT03218683).

S63845 is a highly selective and potent small-molecule inhibitor that binds to the BH3-binding grooves of Mcl-1 and similarly induces apoptosis through interrupting the binding of Mcl-1 to Bak or promoting the release of BIM, resulting in induction of apoptosis through the intrinsic apoptosis pathway (Fig. 2b).^96^ S63845 was demonstrated to be extremely effective in inducing cell death in AML cell lines (IC_50_: 4–233 nM) and primary patient samples, as well as other hematologic and solid malignancies.^96^ MIK665/S64315 belongs to the same series as S63845, though less has been published about its preclinical efficacy. Phase I clinical trials are currently ongoing to establish dosing of MIK665/S64315 in multiple myeloma (MM) (NCT02992483) and in AML patients (NCT02979366) and another is investigating S64315 in combination with venetoclax in AML patients (NCT03672695).

AMG 176 is another potent and selective inhibitor of Mcl-1 that binds to the BH3-binding groove of Mcl-1. It has a picomolar affinity for Mcl-1 and was demonstrated to disrupt the interaction between Mcl-1 and Bak, inducing apoptosis in AML cell lines (Fig. 2b).^99^ AMG 176 is an orally administered molecule with superior PK properties, making it an ideal candidate for in vivo studies. In AML mice xenograft studies, AMG 176 had a dose-dependent reduction in tumor burden when delivered twice weekly.^99^ Several studies have demonstrated that normal B cells, monocytes, neutrophils, and hematologic progenitor cells depend on Mcl-1, so further studies into the tolerability of AMG 176 included using human Mcl-1 knock-in mouse models to look at the effect of AMG 176 on normal hematopoietic cells. AMG 176 was found to reduce numbers of B cells, monocytes, neutrophils, eosinophils, basophils, and reticulocytes in both the peripheral blood and BM in a dose-dependent manner.^99^ Though cytopenias were seen in Mcl-1 knock-in mouse models, there was no systemic toxicity as demonstrated by maintenance of body weight. There is an ongoing phase I clinical trial in R/R MM or AML patients to determine the safety, tolerability, pharmacodynamics, and PK of AMG 176 (NCT02675452). In addition, there is an ongoing phase I clinical trial looking at the combination of AMG 176 with venetoclax in R/R AML and non-Hodgkin’s lymphoma (NHL)/diffuse large B cell lymphoma (DLBCL) patients (NCT03797261). Though preclinical studies demonstrated oral availability, both clinical trials are for IV AMG 176.

Lastly, AMG 397 is an oral small-molecule inhibitor of Mcl-1 and is the only orally available Mcl-1 inhibitor in a phase I trial for MM, AML, NHL, or DLBCL (NCT03465540), though the FDA placed this trial on hold due to concerns for cardiac toxicity and the pharmaceutical company, Amgen, also suspended clinical trials with AMG 176 as a precaution. The cardiac toxicity seen in patients being treated with AMG 397 represents a considerable concern for heart failure as a side effect of Mcl-1 inhibition and will need to be addressed if direct Mcl-1 inhibition is to be utilized clinically.

#### Indirect targeting of Mcl-1

Cyclin-dependent kinase 9 (CDK9) belongs to a family of 13 protein kinases that are transcriptional regulators and are important targets for anticancer activity, and several CDK9 inhibitors are currently under clinical development for the treatment of hematologic malignancies, including AML.^103^ CDK9 is a serine/threonine kinase that forms the catalytic core of the positive transcription elongation factor b (P-TEFb), which is critical for stimulating transcription elongation of most protein-coding genes, including Mcl-1.^104^ CDK9 inhibitors induce apoptosis by preventing the transcription of Mcl-1, leaving Bak and Bax able to oligomerize (Fig. 2c). Though it is a promising target, many CDK9 inhibitors lack specificity and also target other CDKs, resulting in significant off-target effects and toxicity.^103^ Some of the first P-TEFb/CDK9 inhibitors, BAY 1143572 (atuveciclib) and BAY 1251152, were entered into clinical trials for advanced hematologic malignancies but failed to establish safety or efficacy to move to phase II clinical trials (NCT02345382, NCT02745743). Current clinical trials are ongoing for several CDK9 inhibitors, including alvocidib (flavopiridol; NCT03563560, NCT03441555, NCT03969420), dinaciclib (NCT03484520), voruciclib (NCT03547115), and AZD4573 (NCT03263637).

Alvocidib is a flavone molecule that is a pan CDK inhibitor which has demonstrated success in targeting CLL and is currently in clinical trials for AML. Clinical trials in CLL have demonstrated that toxicity is a limiting factor with many patients experiencing serious side effects such as tumor lysis syndrome in addition to lower bioavailability due to high plasma protein binding.^105^ A phase II clinical trial investigated alvocidib, cytarabine, and mitoxantrone (combination termed FLAM) vs cytarabine and daunorubicin (7 + 3 therapy) in newly diagnosed AML patients with non-favorable-risk cytogenetics. FLAM led to significantly increased CR rates compared to 7 + 3 therapy (70% vs 47%) with one cycle, but there was no significant difference in OS or event-free survival.^106^ Further clinical trials are needed to determine the role of alvocidib in combination therapy. Current clinical trials are investigating the combination of alvocidib and venetoclax as a method of treatment that may limit toxicity effects seen in alvocidib monotherapy (NCT03969420).

Dinaciclib, a small-molecule inhibitor of CDK 1, 2, 5 and 9, has been shown to downregulate Mcl-1 and has activity in hematologic malignancies, including AML.^107^ It is currently in several clinical trials for both solid and liquid tumors, including a phase I clinical trial in combination with venetoclax for R/R AML (NCT03484520). AZD4573 is a selective CDK9 inhibitor that has been shown to be successful in treating AML (as well as other hematologic malignancies) both in vitro and in vivo, as demonstrated in cell line-derived and patient-derived xenograft models.^108^ AML cell death was dependent on downregulation of Mcl-1 by inhibition of CDK9 and showed significant synergy when combined with venetoclax.^108^ There is a phase I clinical trial ongoing to test the safety and tolerability of AZD4573 in a variety of hematologic malignancies, including AML (NCT03263637). Another second-generation selective CDK9 inhibitor, voruciclib, was demonstrated to downregulate Mcl-1 expression in AML cells and synergize with venetoclax both in vitro and in mouse xenograft models, demonstrating promising potential as a combinational therapy.^109^ A phase I escalation study is currently recruiting patients with B cell malignancies and AML to determine the safety and preliminary efficacy of voruciclib after treatment with standard therapy (NCT03547115).

Another recent study utilized inhibition of Exportin 1 (XPO1) to decrease expression of Mcl-1.^110^ XPO1 is a nuclear exporter that is overexpressed in AML cells and its inhibition has been correlated with decreased expression of Mcl-1.^111^ This study found that inhibition of XPO1 via Selinexor (KPT-330) reduced Mcl-1 expression and the combination of selinexor and venetoclax were found to be synergistic in AML cell lines and primary patient samples.^110^ There is currently a phase Ib trial of selinexor in combination with venetoclax for AML, DLBCL, and NHL (NCT03955783).

#### Combined targeting of Mcl-1 and Bcl-2

As we have outlined above, both Bcl-2 and Mcl-1 have been found to be important regulators of cell survival in AML and thus have been the focus of development of new targeted therapies. The development and implementation of Bcl-2 inhibitors has uncovered mechanisms of resistance in AML cells, namely, through the upregulation and overexpression of Mcl-1 and Bcl-xL.^82,95,112–115^ To this fact, Pei et al. has recently shown that monocytic subpopulations of AML are inherently resistant to venetoclax due to loss of Bcl-2 expression and reliance on Mcl-1.^116^ In both AML cell lines and primary patient samples, the sensitivity to venetoclax is inversely correlated with the ratio of *Mcl-1*/*Bcl-2* transcripts.^117^ Further studies have demonstrated that Bim released after venetoclax exposure was then sequestrated by Mcl-1, conferring intrinsic resistance to venetoclax in AML cell lines and primary patient samples.^84^ Given that this resistance mechanism exists and understanding that antiapoptotic Bcl-2 family proteins are functionally redundant, there have been many studies showing that dual inhibition of Bcl-2 and Mcl-1 shows promising results in AML and highlight the possibility of circumventing venetoclax resistance and enhancing its antileukemic activity.^97,118–122^

Both direct and indirect inhibition of Mcl-1 has been found to be successful in enhancing venetoclax efficacy in AML. Synergistic effects have been observed with venetoclax in combination with direct Mcl-1 inhibition.^122^ This has led to combination studies with AZD5991 (Mcl-1 inhibitor) and venetoclax, which have shown efficacy in vivo, and authors demonstrated the combination could overcome therapy resistance.^97^ In a study of Mcl-1 inhibitor AMG 176, in combination with other approved AML therapies, authors found that the combination of AMG 176 and venetoclax worked synergistically and completely reduced tumor burden in mice.^99^ As mentioned above, MIK665/S64315 is a highly selective Mcl-1 inhibitor. It has been shown to have potent activity against primary AML cells when used in combination with Bcl-2 inhibitors, including cells with adverse cytogenetic abnormalities and categorized for poor-risk outcomes.^118^ The combination therapy has also been successful in reducing viability of leukemia progenitor cells and LSCs in in vitro and in vivo models, further demonstrating its clinical potential.^118^

In studies of indirectly inhibiting Mcl-1, Choudhary and colleagues demonstrated that downregulation of Mcl-1 with phosphoinositide-3 kinase (PI3K)/mammalian target of rapamycin (mTOR) inhibitors potentiated the effect of venetoclax in leukemia cell lines.^115^ Similarly, downregulation of Mcl-1 via the PI3K/histone deacetylase (HDAC) dual inhibitor, CUDC-907, worked synergistically with venetoclax to induce apoptosis in AML cell lines and primary patient samples.^123^ Preclinical studies have also shown that venetoclax can be used in combination with other approved therapies that downregulate Mcl-1 in AML cells. FLT3 inhibitors, midostaurin and gilteritinib, were demonstrated to strongly enhance the antileukemic activity of venetoclax in *FLT3*-mutated AML cell lines, primary patient samples, and mouse xenograft models, at least partially through downregulation of Mcl-1.^124^

These preclinical studies have demonstrated the powerful combination of Mcl-1 and Bcl-2 inhibition in AML. This has led to several combinational clinical trials that are currently ongoing. These include: a phase I trial assessing AZD5991 + venetoclax (NCT03218683), a phase I trial assessing AMG 176 + venetoclax (NCT03797261), and a phase I trial assessing MIK665/S64315 + venetoclax (NCT03672695). In addition, several of the CDK inhibitors are currently in phase I clinical trials for combination therapy with venetoclax—including alvocidib (NCT03441555, NCT03969420) and dinaciclib (NCT03484520). There is also a phase I clinical trial of venetoclax in combination with gilteritinib in patients with R/R AML and an *FLT3* mutation (NCT03625505).

### Receptor tyrosine kinase (RTK) signaling pathways in AML

**Fig. 3:**
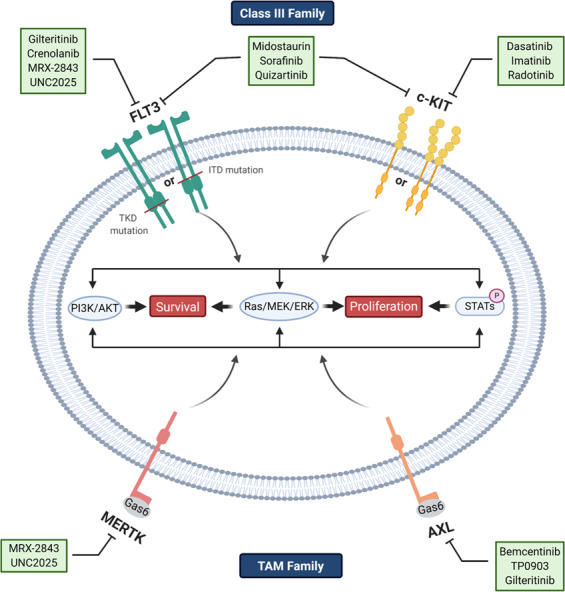
Inhibition of RTKs in AML. Class III (FLT3 and c-Kit) and TAM (MERTK and AXL) RTKs are implicated in leukemogenesis and progression of AML. RTKs support proliferation and survival through PI3K/AKT, Ras/Ref/MEK/ERK, and JAK/STAT signaling pathways. c-Kit is commonly overexpressed in AML and FLT3 mutations in the tyrosine kinase domain (TKD) or ITD in the juxtadomain result in constitutive activation. MERTK and AXL are overexpressed in AML as is their ligand Gas6. Inhibition of RKTs (via inhibitors listed) inhibits downstream signaling and suppresses proliferation and survival of AML cells

RTKs are important in regulating pathways for growth, differentiation, adhesion, and cell death and are implicated in the development and progression of cancer.^125^ RTKs consist of 20 different subfamilies, including class III and TAM family RTKs (Fig. 3). Class III RTKs have been found to have a major impact on leukemogenesis and transformation into AML and include c-Kit, CSF1R, FLT3, and platelet-derived growth factor receptor (PDGFR; Fig. 3). In leukemia, class III RTKs have been associated with aberrant activation that leads to proliferation. Specifically, both *c-Kit* and *FLT3* mutations and expression are important in AML and both are associated with worse prognosis and both RTKs have been important targets in antileukemic therapy development (Fig. 3). The TAM family of RTKs includes TYRO3, AXL, and MERTK, and these are required for normal hematopoiesis of several innate immune cells, vital for platelet activation and stabilization, and has been implicated in erythropoiesis.^126^ In addition to having important functions in normal hematopoietic development, TAM RTKs play an important role in activating proliferation and survival pathways in cancer cells, especially in acute leukemia.^126^ Overexpression or aberrant activation of all three of these TAM RTKs, especially AXL and MERTK, have been associated with hematologic malignancies and have become of interest as targets in the development of new therapies (Fig. 3).^126–130^

There has been an extreme effort in the development of TKIs against a variety of cancers, including hematologic malignancies such as AML.^131,132^ These are classified as type I, II, and III inhibitors, with type I inhibitors binding to ATP pocket of an active protein kinase domain; type II inhibitors bind to an inactive protein kinase domain; type III inhibitors bind to allosteric sites that are not part of the active site.^44,133^ Though there are limitations in specificity, targeting RTK pathways is of extreme importance in AML and both preclinical and clinical studies have demonstrated promising results thus far.

#### FLT3 inhibitors

FLT3 is a class III RTK that is expressed on the cell membrane of early hematopoietic stem cells (HSCs) and progenitor cells. Under normal conditions, it regulates the proliferation and differentiation of these cells when its ligand, the cytokine Fms-like tyrosine kinase 3 ligand (FL), triggers homodimerization of the receptor, conformational changes, and transphosphorylation of its intracellular domains. When bound, FLT3 synergizes with other cytokines (stem cell factor (SCF), interleukin (IL)-3, granulocyte-macrophage colony-stimulating factor, etc.) to promote the generation of myeloid cells.^134^ Downstream effectors of wild-type-FLT3 (wt-FLT3) include the signal transduction pathways PI3K/AKT and Ras/mitogen-activated extracellular signal-regulated kinase (MEK)/extracellular signal–regulated kinase (ERK) (Fig. 3). Interestingly, knockout of this receptor has little effect in hematopoiesis, other than defective repopulation capacities of BM cells upon transplantation.^135^ This means that active FLT3 signaling is not an absolute requirement for normal blood maintenance, but when it is conversely overactivated in malignant hematopoiesis, by mutations to the *FLT3* gene or overexpression, it is frequently a considerable aggressor for AML cell proliferation and survival.

The FLT-3 receptor has five immunoglobulin domains in its extracellular segment, a transmembrane domain, and two TKDs attached to a juxtamembrane (JM) domain.^134^ Of all AML cases with *FLT3* mutations, 25% contain ITD mutations in the JM domain of FLT3, varying in length (3–>400 base pairs), and represent an independent prognostic factor.^136^ *FLT3-ITD* mutations are associated with increased risk of relapse and an inferior OS probability when treated with standard chemotherapy.^14,137^ An additional 7–10% of patients have point mutations in the TKD, but the prognostic role is more unclear for these patients.^137–139^ Both *FLT3-ITD* and *FLT3-TKD* mutations constitutively activate FLT3 kinase activity, resulting in proliferation and survival of AML cells (Fig. 3).^43^ The mutated FLT3-ITD RTK can dimerize with another FLT3, causing the activating phosphorylation of its kinase domains without instigation by FL (Fig. 3). Constitutively active FLT3 not only induces Ras/Raf/MEK/ERK and PI3K/AKT signaling but also phosphorylates signal transducer and activator of transcription factor 5 (STAT5) directly, independent of Janus-activated kinase 2 (JAK2),^140^ but these effects appear to be cell context dependent.^134,137^ The role of FLT3-ITD in leukemogenesis was observed in a FLT3-ITD knock-in mouse model with the insertion of 18 bp in the JM domain. MPNs were generated in this mouse model and examination of the MPN-initiating cells revealed that they originated from long-term HSCs (LT-HSCs). With constitutive activation of FLT3 in these cells, the LT-HSC compartment in the BM experience increased cell-cycle entry and ultimately exhaustion of the LT-HSC population. Treatment with a first-generation FLT3 inhibitor, sorafenib, restored the HSC compartment in these mice and ablated MPNs, demonstrating the efficacy in targeting FLT3 as a therapeutic means.^141^ However, it still stands that AML is a heterogeneous disease, and in fact, untreated AML is frequently a polyclonal disease, where the FLT3 mutation is found in only a subset of the bulk AML population.^142^ Additionally, FLT3 inhibitors have been shown to have off-target effects, and the toxicity may limit efficacy when translating to clinical care.^44^

R/R AML has a larger FLT3 allelic burden or oncogenic modifications, as opposed to its newly diagnosed counterpart and increased dependence on the FLT3-ITD phenotype.^142^ So design of treatment schemas for AML patients with *FLT3* mutations that incorporate FLT3 inhibitors depends on many factors of their disease. These factors include the severity and stage of the disease as well as any other gene mutations collaborating with the FLT3 mutation. The use of FLT3 inhibitors that also target other non-FLT3 kinases such as sorafenib and midostaurin may be ideal due to their ability to target broader populations of AML cells (Fig. 3). However, due to their unselective pharmacology, off-target toxicity and potency is a concern. Midostaurin has been shown to improve OS in newly diagnosed *FLT3*-mutated AML when used in conjunction with intensive chemotherapy. R/R AML tends to be more significantly driven by FLT3-ITD signaling and should benefit from more selective FLT3 inhibitors.^143^ While this is not totally confirmed in the clinic as of yet, current trials combining these various FLT3 inhibitors with induction chemotherapies in untreated AML may help answer this question.

Gilteritinib is a second-generation, type I FLT3 inhibitor that was FDA approved as a single agent in R/R *FLT3*-mutated AML in November 2018. It also has a non-FLT3 function of inhibiting the AXL RTK, which is known to support FLT3-targeted therapy resistance (see AXL section below; Fig. 3).^44,124,144^ In a phase I/II clinical trial, gilteritinib was investigated as a monotherapy in *FLT3*-mutated and wild-type AML patients and approximately 40% of patients achieved CR, with manageable side effects (NCT02014558).^145^ Additional phase I, II, and III clinical trials are currently being conducted to combine it with various forms of induction therapy (NCT02310321, NCT04027309, NCT02927262) and a phase I/II trial is testing its combination with a programmed death-ligand 1-based immunotherapy, atezolizumab (NCT03730012).^44^

A few other FLT3 inhibitors are currently under clinical evaluation, such as crenolanib (NCT01657682) and quizartinib (AC220) (Fig. 3), in combination with clinically approved therapeutics (NCT03552029, NCT02668653, NCT03735875). Quizartinib was approved by the Ministry of Health in Japan for the treatment of R/R FLT3+ AML in 2019 but was rejected by US FDA due to concern over modest survival benefits and the risk for a cardiac disorder. If optimized and approved, these novel agents represent possibilities for a standard option for AML patients with FLT3-ITD both at initial diagnoses and after relapse. Most likely they will be administered in combination with standard chemotherapy and other molecularly targeted agents since the efficacy of FLT3 inhibitors against AML is less than optimal when administered alone but can significantly enhance the effectiveness of other forms of AML therapeutics when combined.

The transcriptome is significantly altered in *FLT3*-mutated AML cells, in comparison to cells with the wt-*FLT3* phenotype. As a result, the overactivation of the downstream signal transduction pathways (PI3K/AKT, Ras/MEK/ERK, JAK2/STAT5) restrain the expression of transcription factors involved in myeloid differentiation, such as PU1 and C/EBPα, and upregulate the expression of many other genes that support AML cell proliferation.^134^ FLT3-ITD also suppresses the activity of the transcription factor FOXO3a, which is a positive regulator of apoptosis by instigating TRAIL expression.^146^ Through the consequential activation of AKT, FOXO3a receives an inhibitory phosphorylation. However, evaluations of clinical samples have not been able to conclude if an elevated level of phosphorylated FOXO3a is associated with a poor prognosis in *FLT3-ITD* AML patients, but low expression of its tumor suppressive antagonist, FOXM1, does correlate with *FLT3-ITD* and chemoresistance in AML patients.^147^ All in all, further studies are necessary to elucidate the relationship between FLT3-ITD and the FOX protein family to identify markers for FLT3 inhibitor drug response. c-Myc is an example of an upregulated pro-leukemic factor that is a result of FLT3-ITD-specific activation of PI3K/AKT signaling and downstream mTORC1 activation (see c-Myc section below).^11^ This relationship has been demonstrated in preclinical studies which found that Myc network of genes are significantly altered in FLT3-ITD knock-in models.^148^

Unfortunately, clinical responses to single agents that inhibit FLT3 are transient and it is necessary to investigate whether FLT3 inhibitors can be combined with other molecular target therapies in a synergistic manner. We have recently reported the cooperation between venetoclax and gilteritinib in a FLT3-ITD AML cell line-derived xenograft mouse model. In vitro, this combination synergistically induced apoptosis in AML cell lines and primary patient samples. Gilteritinib also downregulated Mcl-1, a commonly overexpressed culprit to venetoclax resistance.^124^ Additionally, this combination is being tested in a phase I clinical trial that is currently recruiting subjects with R/R AML (NCT03625505).

One obvious reason that TKIs are only partially effective for FLT3-ITD AML patients is that they do not sufficiently target LSCs. Li et al. isolated an LSC-enriched cell population (CD34+/CD38−) from FLT3-ITD AML patient samples and found that the NAD-dependent SIRT1 deacetylase protein level is elevated in this population. This was in comparison to cells from the same patient that have non-LSC phenotype (CD47-/CD123-), LSC cells with wt-FLT3, and normal hematopoietic cells. The *FLT3-ITD* mutation was associated with increased sensitivity to SIRT1 inhibition (Tenovin-6, TV6) both in vitro and in vivo. Importantly, SIRT1 acts to deacetylate the p53 protein to inhibit its activity and sensitivity to SIRT1 inhibition was dependent on p53 expression. When TV6 was combined with the TKI, quizartinib (AC220), in a FLT3-ITD patient-derived xenogfaft (PDX) model, primary AML cell engraftment (CD45+/CD33+/FLT3-ITD) was significantly reduced in comparison to quizartinib alone. Since quizartinib monotherapy hardly decreased primary and secondary engraftment in these models, the authors indicate that SIRT1 inhibition enhances the efficacy of the TKI through targeting LSCs.^149^ Therefore, FLT3-ITD AML patients may benefit from SIRT1 inhibition in combination with TKI therapy. However, there are no clinical trials testing this combination yet.

#### c-Kit inhibitors

The c-Kit receptor [CD117 or SCF receptor] is a member of the class III RTK subfamily and is important for the self-renewal and differentiation of HSCs, and loss of function results in defects in hematopoiesis, germinal center development, and pigmentation.^150^ Mutations in *c-Kit* and *FLT3* and rare mutations in *JAK2/3* and *PDGFR* are the only known activating mutations in RTKs in AML.^150^ Mutations in *c-Kit* lead to promotion of cell growth, aberrant signaling, and radiation resistance (Fig. 3).^150^ *c-Kit* mutations occur in 17% of AML patients but occur in an estimated 52% of patients with CBF-AML [patients with t(8;21), t(16;16), or inv (16)] and has been correlated with higher rates of relapse and are considered to be poor prognostic markers in CBF-AML patients who have otherwise favorable prognosis.^150,151^ Though mutations in *c-Kit* occur in only a subset of patients, c-Kit expression is found in 60–80% of AML patient samples and is higher than the expression found on normal hematopoietic blast cells.^150,152^ Given the role that c-Kit plays in activation of cell proliferation and survival in AML cells, the high expression in patient samples offers a unique opportunity to target this pathway.

Though there are no c-Kit-specific inhibitors, several small molecules designed to target RTK have broad spectrum ability to cover c-Kit (Fig. 3). Imatinib mesylate (Gleevec) is a type II inhibitor that inhibits ABL, PDGFR, and c-Kit, and was originally designed to target BCR-ABL, a driver mutation in chronic myeloid leukemia (CML; Fig. 3). Imatinib was the first FDA-approved orally available protein kinase inhibitor (2001) and has led to the development of many more TKIs. In a phase II clinical trial, a combination of CLAG (cladribine, cytarabine, granulocyte colony-stimulating factor (G-CSF)) regimen and imatinib mesylate was investigated in patients with R/R AML. The OS rate was 37% with a median OS of 11.1 months and median progression-free survival (PFS) was 4.9 months. Additionally, CLAG plus imatinib was well tolerated in these patients and showed promising efficacy as a treatment option for AML patients.^153^ Other clinical trials in AML have investigated the use of imatinib in combination with approved therapies including LDAC (with insignificant results) and cytarabine and daunorubicin (demonstrating effectiveness) and investigated high-dose imatinib in c-Kit-positive BCR-ABL-negative patients (with no significant improvement).^154–156^ These studies have demonstrated the effectiveness of using TKIs in combination with 7 + 3 induction therapy in AML.

Though other c-Kit inhibitors exist, none are specific to c-Kit and target other tyrosine kinases as well. These include: sunitinib, ponatinib, axitinib, cabozantinib, dasatinib, lenvatinib, regorafenib, sorafenib, and midostaurin.^133^ Of these, dasatinib and midostaurin have been investigated in the context of AML therapy. Midostaurin is thought to be primarily a FLT3 inhibitor (Fig. 3). In preclinical studies of dasatinib in combination with another TKI, radotinib, AML cell death was induced in a c-Kit-dependent mechanism, demonstrating its potential as a therapy in AML (Fig. 3).^157^ In a phase Ib/IIa study, dasatinib was given to newly diagnosed CBF-AML patients following standard induction therapy with daunorubicin and cytarabine and consolidation therapy with high-dose cytarabine. The rate of CR/CRi was 94% with the 4-year estimated OS rate of 74.7%.^158^ In addition, there were no unexpected toxicities with this therapy and the combination was well tolerated. A phase III trial is ongoing (NCT02013648).

#### TAM family inhibitors

As stated above, the TAM family of RTKs consists of TYRO3, AXL, and MERTK, with AXL and MERTK being the most pursued targets of antileukemic therapies for AML (Fig. 3).^159^ A study found that MERTK is overexpressed in almost all AML cell lines and AML patient samples (80–100%) and was an important contributor to leukemogenesis in AML.^127^ The study also demonstrated that activation of MERTK was correlated to activation of downstream pathways that support AML cell survival, including ERK1/2, AKT, and p38, and subsequent inhibition of MERTK in AML cell lines resulted in decreased cell proliferation and colony-forming ability and reintroduction could rescue cell viability (Fig. 3). These in vitro studies translated to prolonged survival in murine xenograft models with MERTK knockdown and demonstrate that MERTK is a promising target in AML.^127^

In parallel with considerations of targeting TAM in AML, TAM kinases are especially important in prosurvival signaling under conditions of stress and can potentially promote cancer cell survival during leukemia therapy (Fig. 3).^159^ GAS6, an upstream activation factor of the TAM family, binds to AXL and MERTK with high affinity and is a common ligand for all three TAM RTKs (Fig. 3). GAS6 has also been shown to be aberrantly expressed in AML cell lines and studies have found that high expression was correlated with worse prognosis.^160^ Both GAS6 and AXL have been associated with the development of resistance in AML cells in response to TK (FLT3) inhibitors and represent an important consideration for therapy development.^144,161^ To this effect, AML patients with *FLT3-ITD* mutations can initially have a good response to FLT3 inhibition but frequently develop resistance.^43^ This has led to the development and success of dual MERTK and FLT3 inhibitors in AML.

UNC1666, a novel MERTK and FLT3 targeted small-molecule TKI, demonstrated antileukemic activity against AML cells. UNC1666 is a type I inhibitor that was found to inhibit both MERTK and FLT3 equipotently and at low concentrations (0.16, 0.67 nM). Though UNC1666 was able to reduce prosurvival signaling downstream of MERTK and FLT3 and induced apoptosis in AML cell lines, it showed poor bioavailability in preclinical studies.^162^ This led to the development of MRX-2843, a small-molecule inhibitor of both MERTK and FLT3 with improved bioavailability compared to UNC1666.^163^ MRX-2843 has demonstrated significant induction of AML cell death that is proportionate to inhibition of MERTK phosphorylation and downstream signaling pathways and is dose dependent in AML cells (Fig. 3). It has demonstrated therapeutic activity in MERTK and FLT3-dependent cell line xenograft models and PDX models, showing promising therapeutic potential.^163^ It also has activity against primary AML patient samples while not affecting normal hematopoietic cells. Importantly, cell lines that were resistant to other FLT3 inhibitors demonstrated sensitivity to MRX-2843 both in vitro and in murine xenograft models.^163^ MRX-2843 is currently in a phase I dose escalation clinical trial to determine safety, PK, and pharmacodynamics in R/R advanced and/or metastatic solid tumors (NCT03510104).

Another promising therapy has come with UNC2025, a selective MERTK/FLT3 inhibitor that was modified from the MERTK inhibitor UNC1062 to be orally bioavailable and has activity against AML cell lines (Fig. 3).^164^ In preclinical studies of UNC2025, authors demonstrated that it was able to inhibit MERTK phosphorylation in BM leukemia cells, induce AML cell death, and decrease colony-forming potential and tumor cell proliferation. It also had efficacy against both cell line and patient-derived murine xenograft models, even when there was significant disease burden at the onset of treatment, and UNC2025 was well tolerated in mice.^165^ Inhibition of MERTK with UNC2025 resulted in dose-dependent increases in survival and was enhanced when UNC2025 was used in combination with methotrexate, an approved chemotherapeutic antimetabolite.^165^ Notably, mice experienced less toxicity compared to other FLT3 inhibitors and hematopoietic defects were described to be reversible. This study demonstrated that dual inhibition of MERTK/FLT3 was effective and well tolerated in preclinical in vivo studies and has potential to be effective in enhancing cytotoxicity of other therapies when used in combination.^165^ There are no current clinical investigations on UNC2025.

AXL expression has been observed in 35% of AML patient samples and has been correlated with shorter OS in AML patients.^128,129^ Inhibition of AXL in AML cells via bemcentinib (BGB324), a selective AXL inhibitor (Fig. 3), inhibited cell survival regardless of FLT3 status and promoted sensitivity to cytarabine and doxorubicin and inhibition showed promising effects in mice with FLT3-ITD AML xenografts.^129^ Based on efficacy in preclinical studies, bemcentinib was entered into several clinical trials. This includes a phase II trial to assess the efficacy of bemcentinib in AML and MDS patients with R/R disease (NCT03824080). Additionally, a phase Ib/II study to investigate dose escalation of bemcentinib and efficacy of bemcentinib in combination with cytarabine or decitabine in patients with AML who are unsuitable for intensive therapy (NCT02488408). In October of 2019, bemcentinib received fast-track designation from the FDA for the treatment of elderly patients with R/R AML based on unpublished data supporting its efficacy in phase II trial (NCT03824080). Bemcentibib is also being studied for efficacy in many types of solid tumors, which are not covered in this review.

TP0903 represents another AXL inhibitor that has had efficacy against AML in preclinical studies and specifically demonstrated the ability to resensitize FLT3 inhibitor-resistant cells (Fig. 3).^144^ TP0903 also has demonstrated activity against CLL and was started in a phase I clinical trial to determine safety and efficacy both as a monotherapy and in combination with ibrutinib in patients with CLL and small lymphocytic lymphoma. This trial was terminated with no report of results (NCT03572634). There are no current clinical trials planned in AML, but there is a phase I trial ongoing in advanced solid tumors (NCT02729298).

Further development and investigation into TAM inhibitors is ongoing and include extensive development in biologic inhibitors, such as mAbs, though these have mostly been investigated in solid tumors.^159^ The combinational use of TAM inhibitors with current cytotoxic therapies and the dual inhibition of TAM and FLT3 pathways to combat therapeutic resistance in AML are promising and offer a way to expand current treatment options.

### HH signaling pathway

HH proteins are important in signaling pathways that promote embryogenesis, maintain adult stem cells, and regulate cell proliferation and differentiation.^57,166^ The HH family consists of three ligand homologs: Sonic HH (SHH), Indian HH, and Desert HH, and these HH proteins bind to the transmembrane proteins Patched-1 (PTCH-1) and Patched-2 (PTCH-2), which act as continuous suppressors of the SMO transmembrane protein.^166^ The interaction of HH and PTCH proteins leads to the disinhibition of SMO, which then phosphorylates and activates glioma (GLI) family zinc finger activating transcription factors (GLI1/2).^57,166^ The activation of GLI1/2 leads to the expression of HH pathway-related genes and are responsible for the induction of cell cycle, antiapoptotic mechanisms, and cell differentiation. The HH pathway is important to normal hematopoiesis and is both lineage and stage dependent and disruption of the HH pathway has been found in many hematologic malignancies, with a strong relation to MDS and progression into AML.^57,167,168^ Studies have found that myeloid malignancies express higher levels of HH proteins and their downstream targets with the majority of AML patient samples overexpressing SHH, PTCH-1, SMO, and/or GLI family proteins.^169^ Importantly, GLI1 expression has been found to be elevated in the blasts of relapsed AML patients. Upregulation of GLI1 and other HH proteins has been correlated with chemoresistance in AML and GLI1 expression is related to worse OS in patients.^170,171^ Subsequent studies have found that inhibition of GLI1 is able to induce cell death and differentiation in leukemic cells, demonstrating the potential for therapeutic success.^168,172^ Additionally, the maintenance of LSCs has been shown to be dependent on HH signaling and inhibition of SMO pathway can target this population of cells, highlighting the role targeting HH may play in preventing relapse in AML.^173^

#### HH pathway inhibitors

This apparent reliance on the HH pathway in leukemia cells has led to investigations into its inhibition. Targeting the HH pathway via SMO inhibitors was shown to sensitize resistant cells to cytarabine and doxorubicin.^174^ Preclinical studies of erismodegib (LDE225) and vismodegib (GDC0449), SMO inhibitors, found that inhibition of SMO improved efficacy of doxorubicin and azacitidine both in vitro and in AML xenograft mouse models.^174–176^ These SMO inhibitors were further studied in clinical trials (erismodegib: NCT01826214, and vismodegib: NCT01880437), but vismodegib was terminated early due to lack of efficacy and neither agents showed promising results as monotherapies. Both vismodegib and erismodegib showed dose-limiting toxicities in clinical trials, but another SMO inhibitor, glasdegib, has a much shorter half-life and was well tolerated at lower doses. The shorter half-life of glasdegib could contribute to lower toxicity to normal stem cells and improved ability to target LSCs, especially in combination with other therapeutic agents.^177^

Glasdegib (PF-04449913) is a selective, small-molecule inhibitor of SMO that has demonstrated efficacy against AML cells both in vitro and in vivo in PDX mouse models.^178^ In this study, glasdegib was able to attenuate leukemia-initiation potential in serial transplant mouse models and sensitized AML cells to azacitidine.^178^ Glasdegib entered clinical trials, and a phase II study investigated the efficacy of glasdegib in combination with current standard AML therapies (NCT01546038). In this trial, patients received glasdegib (100 mg/day) with LDAC (20 mg subcutaneously, twice daily on days 1–10 of 28-day cycle) or LDAC alone.^59,179^ The combination therapy had a median OS of 8.3 months and was significantly improved compared to the LDAC alone group, which had a median OS of 4.3 months.^59,179^ In AML patients, glasdegib plus LDAC showed a significant improvement in CR rate compared to LDAC alone (17 vs 2.3%) and OR was also higher in glasdegib plus LDAC compared to LDAC alone (26.9 vs 5.3%).^59,179^ In this study, the combination therapy was generally well tolerated and patients had similar rate of serious adverse effects reported compared to LDAC alone, with the most frequent being febrile neutropenia and pneumonia and adverse effects usually related to SMO inhibitors (alopecia, muscle spasms, and dysgeusia) were less frequently reported.^179^ In the same phase II clinical trial (NCT01546038), another arm evaluated glasdegib plus cytarabine/daunorubicin in patients with untreated AML or high-risk MDS. Patients received glasdegib (100 mg orally) once daily for 28 days, with cytarabine (100 mg/m^2^ IV) on days 1–7 and daunorubicin (60 mg/m^2^ IV) on days 1–3 (standard 7 + 3 induction), followed by consolidation therapy consisting of cytarabine (1 g/m^2^ twice daily, on days 1, 3, and 5 of each cycle for 2–4 cycles) and maintenance therapy consisting of glasdegib for a maximum of 6 cycles. In patients aged ≥55 years, 40% achieved CR and in patients aged <55 years, 88.9% achieved CR and median OS for all patients was 14.9 months.^180^ The combination of glasdegib plus cytarabine/daunorubicin was well tolerated and similar safety profile to those receiving intensive chemotherapy and to glasdegib plus LDAC combinations.^180^ These studies have demonstrated that inhibition of the HH pathway can improve efficacy of current AML therapies and improve patient outcomes. Following these results, glasdegib became the first HH pathway inhibitor to be FDA approved (November 2018) to be used in combination with LDAC for newly diagnosed AML patients who are aged ≥75 years or for those who have comorbidities that preclude intensive induction therapy.^59^ In a long-term follow-up (42–43.4 months) to this phase II BRIGHT AML trial (NCT01546038), authors reported that newly diagnosed AML patients who were ineligible for intensive chemotherapy continued to demonstrate improved OS when treated with glasdegib + LDAC vs LDAC alone. However, deaths due to disease progression were similar across groups and both groups experienced a high percentage of patients who did not respond (glasdegib + LDAC, 43%; LDAC alone, 33%).^181,182^ Though approved for use, it is still unclear the beneficial role that glasdegib has in targeting LSCs in combination therapy in elderly patients, especially in comparison to venetoclax + HMA, which is the current standard of therapy.

Currently, glasdegib is being studied in several different clinical trials in combination with other therapeutics in AML. This includes a phase II study to evaluate glasdegib in combination with decitabine in newly diagnosed poor-risk AML patients who are ineligible for intensive therapy (NCT04051996). Similarly, a phase I study in Japanese patients is investigating glasdegib as a single agent or in combination with LDAC or cytarabine and daunorubicin in previously untreated patients with AML or high-risk MDS or in combination with azacitidine in previously untreated patients with AML (NCT02038777). A phase II study is underway to determine the efficacy of CPX-351 (liposomal formulation of cytarabine and dauorubicin) in combination with glasdegib in patients with AML with MDS-related changes or therapy-related AML (NCT04231851). A phase Ib study is evaluating the safety, efficacy, PK, and pharmacodynamics of glasdegib in combination with azacitidine in patients with untreated high-risk MDS, AML, or chronic myelomonocytic leukemia (NCT02367456). Another combinational phase Ib/II trial is set to determine the safety and dosage of anti-OX40 antibody (immunotherapy that promotes T cell response) in combination with venetoclax, avelumab, glasdegib, gemtuzumab ozogamicin, and azacitidine in patients with R/R AML (NCT03390296). And lastly, a phase III trial is ongoing to determine efficacy of gemtuzumab ozogamicin (ADC for CD33-directed antibody) and glasdegib in older patients with newly diagnosed AML (NCT04093505).

### Mitochondria pathway targets

**Fig. 4:**
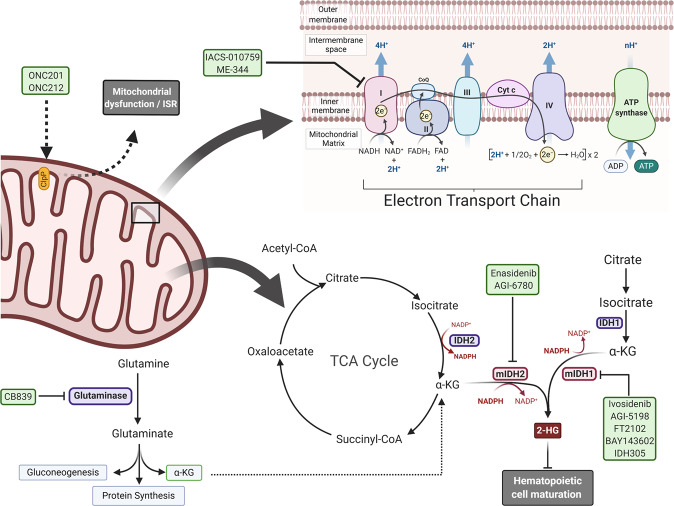
Targeting AML cell mitochondria function. Expression of gain-of-function mutant IDH1/2 (mIDH) results in the production of oncometabolite, 2-HG, which inhibits maturation of hematopoietic cells and promotes leukemia. Inhibitors specific to mIDH (enasidenib, AGI-6780, ivosidenib, AGI-5198, FT2102, BAY143602, IDH305) prevent the production of 2-HG and promote differentiation of leukemia cells. CB893, a glutaminase-specific inhibitor, prevents the production of α-ketoglutarate (α-KG)—a key metabolite for mIDH cell survival. IACS-010759 and ME-344 target AML cell OXPHOS through inhibition of ETC complex I. ONC201/ONC212 induce mitochondrial stress through activation of mitochondrial protease, ClpP, which leads to subsequent mitochondrial dysfunction and AML cell stress (ISR)

Cancer cells utilize metabolic adaptations and deregulation of cellular energetics that support cell growth and division. They can do this not only through increased dependence on glycolysis, the Warburg effect, but also can modify mitochondria through induction of changes to mitochondrial DNA (mtDNA), increased reactive oxygen species (ROS) production, and changes in oxidative phosphorylation (OXPHOS).^183,184^ As there is a greater understanding about the dynamic role that mitochondria play in cancer cell survival and persistence, the development of therapeutics to target mitochondria (mitocans) has been of increasing interest.^183^ There are a diverse amount of pathways that can be targeted to impact mitochondria, including apoptosis (Bcl-2 family proteins), enzymatic pathways [tricarboxylic acid (TCA) cycle, glutaminolysis, fatty acid synthesis, etc.], ROS generation, and mitochondrial respiration as only a few examples.^183^ Extensive studies have shown that AML demonstrates an increased dependency on mitochondria.^185^ Notably, AML cells have associated mutations in mtDNA, reportedly higher mtDNA copy numbers, are dependent on OXPHOS for ATP production (especially in LSCs and IDH-mutated AML cells), and utilize induction of mitochondrial changes to facilitate resistance against treatments.^186–190^ These studies both demonstrate AML dependence on mitochondria for survival and persistence and provide context for the need for therapies that target this vulnerability in AML. Here we outline both preclinical and clinical investigations into mitochondrial pathway therapies in AML (Fig. 4).

#### ***IDH*mutations in AML**

*IDH1* and *IDH2* mutations can be found in up to 20% of AML patients and are one of the more frequently occurring mutations in cytogenetically normal AML patients.^47^ IDH enzymes are key regulators in cellular metabolism and are also important for adaptation to hypoxia, histone demethylation, and DNA modification.^191^ IDH1 (cytosolic) and IDH2 (mitochondrial) enzymes catalyze the conversion of isocitrate to α-ketoglutarate (α-KG) and produce reduced nicotinamide adenine dinucleotide phosphate (NADPH). IDH2 is a key enzyme in the TCA cycle and IDH1 functions cytosolically to produce α-KG (Fig. 4). Somatic point mutations in *IDH* genes are heterogeneous and cause a single amino acid substitution in the enzymatic active site, resulting in an enzymatic gain of function.^192^ Mutant enzymes catalyze the NADPH-dependent conversion of α-KG to 2-HG, which has been characterized as an oncometabolite that promotes tumorigenesis (Fig. 4).^193,194^ In addition to being an important player in many metabolic pathways, α-KG is an essential cosubstrate for several dioxygenase enzymes that are involved in epigenetic regulation. The depletion of α-KG along with the inhibitory effect of 2-HG in *IDH*-mutated cells leads to changes in epigenetic regulation, notably methylation of regulatory genes, and supports initiation of leukemia in myeloid progenitor cells.^195,196^

Selective inhibitors of IDH mutant enzymes have been heavily pursued, and several inhibitors have been developed so far. Preclinical studies on IDH1 inhibitors (AGI-519) and IDH2 inhibitors (AGI-6780) demonstrated that targeting the mutant enzymes was successful in reducing levels of 2-HG to normal physiologic levels and induces the differentiation of leukemic cells into normal progeny cells.^197,198^ This has led to the development of several IDH mutant inhibitors and the US FDA approval of two since 2018 (Fig. 1).

#### **FDA-approved IDH mutant inhibitors**

Ivosidenib (AG-120) is a small-molecule inhibitor of mutant IDH1 that was developed by Agios Pharmaceuticals and CStone Pharmaceuticals to treat many malignancies with IDH1 mutations, including AML (Fig. 4). In July of 2018, ivosidenib received global approval in the USA for the treatment of adults with R/R AML with an *IDH1* mutation (Fig. 1). In a phase I clinical trial on 125 patients, ivosidenib demonstrated some success as a monotherapy, with ORR of 42% and CR/CRi of 30% with median response time of 8 months. This trial is still ongoing to assess safety, PK, pharmacodynamics, and efficacy (NCT02074839).^50,199,200^ A phase Ib/II study is ongoing to look at the combination of oral ivosidenib and subcutaneous azacitidine for newly diagnosed untreated AML patients with *IDH1* mutations who were ineligible for intensive chemotherapy. Early results from this study demonstrate that combination therapy was associated with ORR of 78% (18/23 patients) and a 44% CR rate (10/23) (NCT02677922).^50^ These results have led to a phase III multicenter, randomized, double-blind, placebo-controlled study, which is ongoing, investigating ivosidenib plus azacitidine in previously untreated *IDH1*-mutated AML patients (NCT03173248).^50^ In another combinational approach, there is a phase I clinical trial investigating the use of ivosidenib in combination with induction (daunorubicin or idarubicin for 3 days with cytarabine for 7 days) and consolidation therapy in *IDH1*-mutated AML patients (NCT02632708). This study is currently ongoing, but initial results showed that 77% (23/30) of patients receiving ivosidenib had achieved CR or CRi.

There are several other clinical trials for ivosidenib that include a phase I trial to investigate efficacy and safety of ivosidenib in combination with fludarabine, cytarabine, and G-CSF (FLAG) chemotherapy in R/R AML and MDS patients with *IDH1* mutations (NCT04250051). There are currently two ongoing phase I trials to test the PK, pharmacodynamics, safety, and clinical efficacy of ivosidenib in patients with R/R AML with *IDH1* mutations (NCT02074839, NCT04176393). In addition to these phase I studies, there is a phase II trial to determine ivosidenib efficacy in *IDH1*-mutated MDS patients (NCT03503409). Lastly, there is an open phase I study to investigate the use of ivosidenib as maintenance therapy for *IDH1*-mutated myeloid neoplasms following allogeneic SCT (NCT03564821).

Enasidenib (AG-221) is a small-molecule inhibitor of mutant IDH2 developed by Celgene Corporation and Agios Pharmaceuticals. Preclinical studies demonstrated that enasidenib promoted AML cell differentiation via inhibition of IDH2 mutants and suppression of 2-HG production (Fig. 4).^201^ The first phase I/II trial of enasidenib is currently ongoing and interim results are promising for efficacy in AML/MDS patients with *IDH2* mutations. Patients were categorized into separate subgroups based on age, R/R status, and prior treatment. The ORR in patients with R/R *IDH2*-mutated AML was 40.3%, CR in 19.3%, and CRi in 6.8% (PR = 6.2%, morphological leukemia-free state = 8.0%). The median duration of response was 5.8 months (8.8 months for CR patients) with OS of 9.3 months (19.7 in patients with CR) and a 1-year survival was estimated to be 39% (NCT01915498).^49,202^ Overall, treatment was well tolerated and these studies have led to enasidenib being the first IDH2 mutant inhibitor to be FDA approved for the use in R/R *IDH2*-mutated AML.^203^ Currently, there is a randomized, open-label, phase III clinical trial comparing enasidenib vs conventional therapeutic regimens in older patients (≥60 years) with R/R *IDH2*-mutated AML following failed treatments (NCT02577406). Additionally, there are ongoing studies investigating the use of enasidenib (100 mg/day) (or ivosidenib, see above) in combination with induction or consolidation therapies (daunorubicin 60 mg/m^2^/day or idarubicin 12 mg/m^2^/day for 3 days with cytarabine 200 mg/m^2^/day for 7 days) (NCT02632708) and the combinational use of enasidenib (100 mg/day) with azacitidine (75 mg/m^2^/day) in newly diagnosed *IDH2*-mutated AML patients (NCT02677922).

There are many other clinical trials with enasidenib currently planned or ongoing. These include a phase II trial in pediatric patients with R/R AML with *IDH2*-mutations (NCT04203316), a phase II trial in R/R AML patients with *IDH2* mutations to investigate whether enasidenib can be used as maintenance therapy post salvage induction therapy (NCT03881735), and two other phase II trials investigating the use of enasidenib in the treatment of MDS as a monotherapy or in combination with azacitidine (NCT03744390, NCT03383575). Enasidenib is also being investigated in two phase I clinical trials as maintenance therapy following allogeneic SCT in *IDH2*-mutated AML patients (NCT03515512, NCT03728335). Other combinational studies include a phase III, double-blind, randomized, placebo-controlled study of ivosidenib or enasidenib in combination with induction and consolidation therapy followed by maintenance therapy in patients with newly diagnosed AML or MDS and aims to follow 968 patients (NCT03839771). Lastly, there is an open phase II study looking at the combination of CPX-351 (liposome-encapsulated cytarabine and daunorubicin) and enasidenib in R/R AML patients with *IDH2*-mutations (NCT3825796).

#### IDH mutant inhibitors in the pipeline

There are several other IDH mutant inhibitors that have been shown to target *IDH*-mutated AML cells (Fig. 4), several of which are currently in clinical trials. FT-2102 is a potent, orally bioavailable IDH1-mutant inhibitor that has been demonstrated to inhibit 2-HG production in xenograft mouse models and has the potential to penetrate the blood–brain barrier.^58^ It is currently in phase I clinical trials in combination with ASTX727 (DNA methytransferase inhibitor) (NCT04013880) and in combination with azacitidine or cytarabine in patients with AML or MDS (NCT02719574). BAY1436032 is a novel oral pan-mutant IDH1 inhibitor, which has had efficacy in vitro and in vivo against AML cells with various *IDH1* mutations. Inhibition of IDH1 mutants with BAY1436032 resulted in differentiation of leukemic cells and leukemic blast clearance that promoted survival in AML PDX mouse models.^204^ A phase I study on safety and preliminary clinical effect of BAY1436032 in *IDH1*-mutated AML patients was completed in April 2019, but no clinical results have been published yet (NCT03127735). IDH305 is another orally bioavailable IDH1 inhibitor with selective inhibition against IDH1-R132H mutant and has shown efficacy in preclinical studies.^205^ In a phase I trial of patients with advanced malignancies with *IDH1* R132H mutations (glioma, AML, MDS, non-CNS solid tumors, and unknown), IDH305 was administered at varying doses twice daily. AML patients had varying response to IDH305, with 33% of them responding, though the trial is currently on hold and there are no official results reported yet (NCT02381886).^197^

#### Other IDH pathway targets

In addition to the epigenetic changes, *IDH*-mutated cells experience major changes to glutamine and TCA cycle metabolism. Glutamine is the main source of α-KG in *IDH*-mutated leukemia cells, leading to a glutamine dependence.^193,206^ Targeting this metabolic dependence through inhibition of glutamine metabolism has been shown to suppress growth of *IDH*-mutated AML cells.^206,207^ CB-839 is an orally bioavailable glutaminase inhibitor that has been demonstrated to reduce levels of 2-HG in *IDH*-mutated AML cells, ultimately promoting the differentiation of leukemic cells (Fig. 4). CB-839 is currently in a phase Ib/II clinical trial in combination with azacitidine in patients with AML/MDS, with evaluations of patients with *IDH*-mutations as an exploration point of the study (NCT03047993).^208^ Solid tumors harboring mutant IDH1 enzymes have also been shown to have an increased ROS production and downregulation of mitochondrial respiration complexes.^209^ *IDH1* mutations have been associated with both increased oxidative metabolism and a sensitivity to inhibition of the mitochondrial electron transport chain (ETC) and OXPHOS suggesting an opportunity for a new therapeutic target in these cells.^210^ Changes to metabolism and mitochondria function also support studies that show how *IDH* mutations induce a cellular dependence on Bcl-2 through a 2-HG-mediated inhibition of cytochrome *c* oxidase (complex IV/COX) in the ETC. Suppression of COX lowers the threshold to trigger apoptosis under Bcl-2 inhibition, and both *IDH*-mutated primary patient cells and AML cell lines have increased response to Bcl-2 inhibitor, venetoclax, in vitro and in PDX models.^211^ This has led to clinical trials where a phase II study found a higher response rate in *IDH*-mutated AML patients with venetoclax monotherapy (33% vs 19%).^55^ Currently, combination therapy is being trialed with venetoclax and IDH1 mutant inhibitor (ivosidenib) in *IDH1*-mutated patients (NCT03471260) and with venetoclax and IDH2 mutant inhibitor (enasidenib) in R/R *IDH2*-mutated AML patients (NCT04092179).

#### ETC inhibitors

Metabolic reprogramming is a well-established hallmark of cancer. The Warburg effect is a phenomenon in which cancer cells utilize glycolysis rather than OXPHOS for ATP production, which in turn adapts tumor cells for higher biosynthesis leading to proliferation. Much effort has been focused on targeting glycolysis. However, OXPHOS is not a common pathway explored for drug targeting, due to gaps in knowledge of the role of OXPHOS in cancer. There are a number of cancers that are reliant on OXPHOS including some subtypes of AML. Previous studies have demonstrated that both AML cell lines and patient samples have higher mitochondrial mass and low spare reserve capacity that renders them more susceptible to oxidative stressors and more sensitive to respiratory chain inhibitors.^186^ Lagadinou et al. found that inhibition of Bcl-2 reduced OXPHOS in AML cells and induced cell death in LSCs, demonstrating both the ability to inhibit OXPHOS and target vulnerable subsets of AML cells.^17^ Below we detail promising OXPHOS inhibitors in AML.

The small-molecule inhibitor of complex I in the ETC, IACS-010759, is currently in a phase I clinical trial for AML (NCT02882321).^212^ IACS-010759 has a unique binding site on complex I of the ETC, compared to other complex I inhibitors (Fig. 4). Photoaffinity labeling experiments have shown that IACS-010759 binds to membrane subunit ND1 of complex I.^213^ Another study also suggested that the complex I-binding site of IACS-010759 is at the ND1 subunit as H292 cell clones containing a Leu55Phe mutation in the ND1 subunit showed lower susceptibility to IACS-010759. This suggests that Leu55, which is located at the proposed ubiquinone access channel, is likely involved in the mechanism of inhibition. However, it has not been clearly identified whether IACS-010759 directly interacts with Leu55.^212^ IACS-010759 was derived from BAY 87–2243, which is an agent that specifically suppressed HIF-1 target gene expression through inhibition of complex I.^213^ Like other complex I and ETC inhibitors studied, BAY 87–2243 was cytotoxic to normal cells, limiting its use in clinical application. BAY 87-2243 failed phase I clinical trials due to dose-limiting toxicities, highlighting the barrier to application of these inhibitors as therapies.^214^ Though IACS-010759 is similar in structure to BAY 87-2243, its mechanism of complex I inhibition differs from that of other quinone-site inhibitors in its class in that it has been suggested to indirectly affect quinone redox reactions by inducing structural changes to the quinone-binding pocket.^213^ It has been proposed that this may contribute to the reduced toxicity observed in in vivo models, and there is hope that this alternative mechanism of action will contribute to an improved safety profile in ongoing clinical studies.

Another mechanism to reduce side effects in normal cells is using ETC inhibitors in combination with other approved anticancer agents. It was recently discovered that AML is more susceptible to drugs that target the mitochondria.^187,188^ IACS-010759 in combination with microtubule destabilizer, vinorelbine, resulted in synergistic induction of apoptosis in AML cell lines and primary patient samples, while normal peripheral blood mononuclear cells were significantly unaffected. In addition, this drug combination inhibited mitochondrial respiration and lowered mitochondrial ATP levels.^188^ Cells from patients with poor prognosis in CLL also showed an increased dependence on OXPHOS. When these cells were treated with IACS-010759, OXPHOS was inhibited and glycolysis was upregulated as a compensatory mechanism. To inhibit both OXPHOS and glycolysis, IACS-010759 and 2-deoxy-d-glucose were used in combination. This combination successfully inhibited both pathways of ATP production and resulted in significant cell death, suggesting that multiple pathways should be targeted to overcome metabolic reprogramming activated in cancer cells.^215^ Metabolic reprogramming toward OXPHOS can also contribute to therapeutic resistance. This is evident in the case of Bruton’s TKI, ibrutinib, which is used for the treatment of mantle cell lymphoma.^216^ Interestingly, IACS-010759 has shown promising effects in ibrutinib-resistant patient-derived in vitro and in vivo cancer models.^217^

A recently discovered complex I inhibitor, ME-344, by MEI Pharma, is the active demethylated metabolite of a synthetic isoflavone NV-128.^218^ NV-128 moderates the degradation of the ETC complex IV subunits, COX I and COX IV. It also induces caspase-independent cell death by activating mitochondrial MAP/ERK kinase pathway and upregulating the proapoptotic protein Bax, leading to the loss of mitochondrial membrane potential.^219^ Through inhibition of mTOR, NV-128 causes the delocalization of EndoG, which cleaves DNA and condenses chromatin.^219,220^ NV-128 has been found to be cytotoxic in several cancer models, though it has yet to be tested in hematological cancers. ME-344 has been shown to be even more effective at inducing cell death at lower concentrations compared to NV-128, making it a promising potential therapy.^220,221^ While the specific cellular target of ME-344 has yet to be confirmed, it induces cell death in solid tumors by interfering with OXPHOS and increasing ROS production. Its efficacy depends on several features of the cancer cells it is in contact with, including the bioenergetic profile and metabolic flexibility.^222^

ME-344 has been extensively studied in preclinical models of solid tumors, and several studies revealed the ability of ME-344 to repress OXPHOS and the ETC (Fig. 4). These studies found that ME-344 is an inhibitor of complex I,^223^ impacts redox homeostasis^224^, and binds to heme oxygenase 1, inhibiting its activity.^225^ Interestingly, one study suggested that ME-344 may be utilized as a mitochondrial inhibitor over metformin since it appears to be more potent and does not need a membrane transporter for cancer cell uptake.^226^ This extensive preclinical data prompted clinical testing of ME-344 in a phase I, open-label, safety, and PK study in 30 patients with refractory solid tumors. It was generally well tolerated with the most common side effects including nausea, dizziness, and fatigue. Some dose-limiting neuropathy was noted but these were in patients who received the drug at above the MTD: 10 mg/kg. At this MTD, a therapeutic index was suggested, with some patients achieving PR or prolonged stable disease. Median PFS was 6.9 weeks in a range of 0–≥52 weeks.^227^ Following the initial clinical study, a phase Ib clinical trial for the treatment of metastatic small cell lung, ovarian, and cervical cancers was initiated to test ME-344 in combination with the chemotherapy drug topotecan (NCT02806817). Early results from this study suggest that this combination did not reach ideal efficacy, but the researchers suggest that combination with an anti-angiogenic TKI will be more successful since the reduction of vasculature by TKIs reduce the rate of aerobic glycolysis and increase mitochondrial respiration.^218,228^ Finally, a most recent randomized phase 0/I trial in HER2-negative breast cancer tested ME-344 in combination with bevacizumab, a mAb against vascularized endothelial growth factor. They investigated biological activity via biopsy analysis taken on days 0 and 29 and found that ME-344 significantly decreased marker of proliferation (Ki67) in patient samples. In addition, they demonstrated that succinate dehydrogenase (SDH; complex II of ETC) expression was significantly elevated in patients receiving ME-344, suggesting that ME-344 does inhibit complex I in cancer cells. These results indicated that ME-344 induced cell death and inflicted its on-target effects in HER2-negative breast tumors.^229^

While ME-344 appears to be a promising novel component for several solid cancer treatment regimens, it has only been tested marginally in AML. Jeyaraju et al. found that ME-344 is cytotoxic to both AML cell lines and primary patient samples but spares normal hematopoietic cells. Importantly, in AML cell line-derived xenograft models, ME-344 suppressed tumor growth by up to 95% of control without evidence of toxicity.^230^ However, they also determined that the cell death imposed by ME-344 was due to the targeting of tubulin. While ROS was increased in the AML cell line OCI-AML2 post ME-344 treatment, concurrent treatment with the antioxidant *N*-acetylcysteine did not rescue the cells. While they did not directly test for mitochondrial respiration suppression in AML cells, these authors demonstrated that molecular mechanisms additional to ROS accumulation exist that contribute to the antileukemic activity of ME-344.^231^ Future studies looking into the molecular mechanism of ME-344 and its efficacy in AML are warranted.

#### Other mitochondrial-targeting drugs

ONC201 is a novel small molecule that is the founding member of the imipridone class of compounds discovered by Oncoceutics, Inc. in a screen for inducers of *TNF-related apoptosis-inducing ligand* (*TRAIL*) gene transcription.^232^ In addition to prompting upregulation and activation of TRAIL to induce apoptosis in a p53-independent manner, ONC201 has been shown to induce an integrated stress response (ISR) and inhibit AKT and ERK, further contributing to induction of cell death in solid tumors.^233–235^ In support of the induction of ISR, ONC201 is a selective antagonist of the G protein-couple receptors (GPCRs), specifically dopamine D2-like receptors (DRD2 and DRD3), and antagonism of the DRD2 receptor promotes cellular toxicity and induction of ISR.^236^ ONC201 has also been shown to kill breast cancer cells through targeting mitochondrial metabolism and, in line with previous studies, authors related inhibition of mitochondria to the activation of ISR and induction of ATF4 and CHOP proteins.^237^ Further investigation into ONC201 found that it is active against hematologic malignancies as a single therapy or in combination with cytarabine or azacitidine in AML cells.^238,239^ Another study by Ishizawa and colleagues found that ONC201 eliminated LSCs and leukemia progenitor cells in AML mouse xenograft models, though the mechanism of action was independent of TRAIL induction and more likely through an ISR pathway.^240^ The mechanism of action was further demonstrated to include activation of ClpP, a mitochondrial protease, in the induction of mitochondria proteolysis in both solid tumors and AML cells.^241,242^ Ishizawa et al. further highlighted that targeting ClpP in AML cells is especially advantageous as it is overexpressed in 45% of primary AML samples (stem cell and bulk populations) and targeting of ClpP leads to dysfunction in mitochondrial respiration and ISR (Fig. 4).^243^ ONC201 is currently being evaluated in clinical trials in many different solid and hematologic malignancies and has been shown to be well tolerated with favorable bioavailability as an oral therapy.^244,245^ Current clinical trials in AML include a phase I/II study in patients with R/R acute leukemias and high-risk MDS (NCT02392572) and a phase I study investigating ONC201 as a maintenance therapy in patients with AML/MDS following allogeneic HSC transplant (NCT03932643).

ONC212 is a new imipridone molecule that is a derivative of ONC201 with increased potency.^246^ It has been utilized in in vivo studies with mouse xenograft models in solid tumors and demonstrated similar efficacy compared to ONC201 even though it showed a decreased serum half-life and was well tolerated as an oral therapeutic.^246^ Similar to ONC201, ONC212 was shown to interact with an orphan GPCR, GPR132, and induced an ISR in AML cells and GPR132 expression in AML cells was positively correlated with sensitivity to ONC212.^247^ Nii et al. further demonstrated that ONC212 induces cell death in patient-derived AML cells but did not affect viability of normal hematopoietic cells and showed efficacy in AML cell line xenograft mouse models over ONC201 at similar doses.^247^ This study also found that the combination of ONC212 and venetoclax in AML cell line xenograft mouse models significantly prolonged survival compared to monotherapy, demonstrating the ability to utilize ONC212 in combination with existing AML therapies. In a final study, ONC212 was confirmed to also activate ClpP and induction of cell death is dependent on this interaction, highlighting its potential use as a mitotoxic drug (Fig. 4).^248^ There are no current clinical trials with ONC212.

### c-Myc-regulated pathways

One of the most notorious oncoproteins in many types of malignancy is the global transcription factor, c-Myc. c-Myc both activates and represses expression of its target genes and is dependent on the availability of a complex system of co-factors that dimerize with c-Myc. When it is dimerized and interacting with basic helix–loop–helix leucine zipper, it will promote the transcription of target genes.^249^ In contrast, c-Myc may also bind to the Myc-interacting zinc finger protein 1 (Miz-1). When bound to c-Myc, Miz-1 acts to inhibit transcription as it is unable to bind to initiator elements of the tumor-suppressor genes (*cdkn2b* [p15] or *cdkn1a* [p21^Cip1/Waf1^]) and thus supports oncogenic cell cycle progression.^250^ The DNA methyltransferase DNMT3a is also recruited by c-Myc in this repression process, which methylates CpG islands of this region and further represses the initiation of transcription.^250,251^

In cancer, c-Myc plays a critical role in preventing apoptosis and promoting drug resistance and supports leukemogenesis.^252–254^ Therefore, targeting of c-Myc is often considered for novel therapeutics of cancer, including AML.^255–258^ This global transcription factor regulates the expression of 15% of all genes, which are involved in seemingly all cellular functions and c-Myc overexpression has been implicated in most subtypes of AML.^259–262^ Further, recent studies demonstrated that c-Myc protein expression is an important prognostic factor in AML.^263,264^ c-Myc also regulates the expression of other transcription factors and miRNAs, emphasizing the control it has over many cellular functions. This supports why many solid and liquid cancers are considered “addicted” to c-Myc, which just so happens to have coined the term for “oncogene addiction.”^251^ In 1999, Felsher and Bishop reported that in hematopoietic cells of transgenic mice the expression of c-Myc leads to lymphoid and myeloid malignancies and that this process was reversible when c-Myc expression was terminated.^265^ Even with concerns of inhibition leading to off-target toxicity, it has continued to develop as a desirable target for AML treatment.

**Fig. 5:**
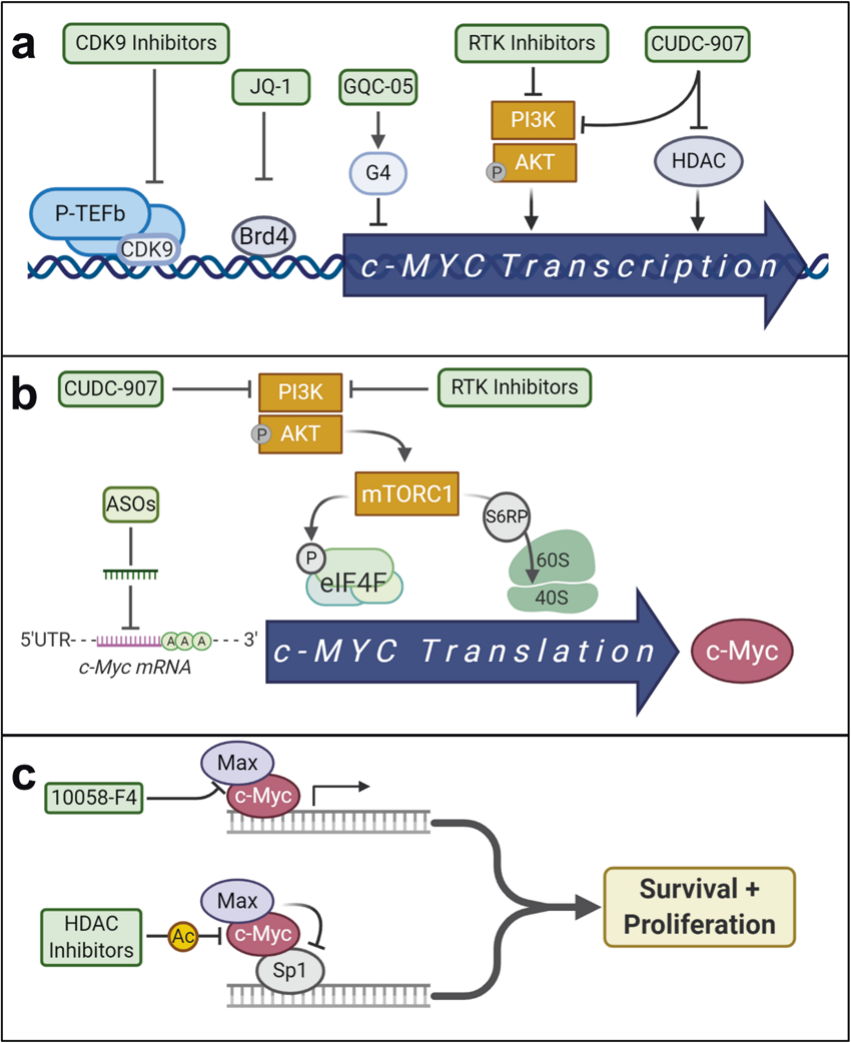
Targeting c-Myc signaling in AML. **a** c-Myc (*MYC*) transcription is promoted through interactions with Brd4 and P-TEFb, which includes CDK9. Targeted therapies that suppress the initiation of transcription include the Brd4 inhibitor, JQ-1, and inhibitors of CDK9. Other inhibitors of c-Myc transcription include the G-quadruplex (G4) ligand, GQC-05, and the dual inhibitor of PI3K and HDAC, CUDC-907. Inhibitors of HDAC may work to inhibit *c-Myc* transcription through HDAC6, which stabilizes a transcription factor that influences c-Myc expression. PI3K/AKT signaling also promotes c-Myc transcription and is inhibited via RTK inhibitors. **b** c-Myc translation is dependent on the formation of the translation-initiation complex, eIF4F, and the assembly of ribosomal subunit, 40S, which are both promoted through the activity of mTORC1. mTORC1 is indirectly suppressed through inhibition PI3K/AKT via RTK inhibitors and CUDC-907. Additionally, c-Myc translation is repressed via ASOs binding to *c-Myc* mRNA transcripts. **c** c-Myc promotes AML cell survival and proliferation and is reliant on interactions with its co-factor Max to promote transcription of survival signals. 10058-F4 inhibits the interaction of c-Myc and Max to prevent transcription and promote AML cell death. c-Myc represses Sp1, a transcription factor, to also promote cell survival. HDAC inhibitors induce acetylation of c-Myc to free Sp1 to promote cell death

There are multiple levels in which c-Myc can be regulated, many of which are by signal transduction pathways. These include PI3K/AKT, MEK/ERK, and JAK/STAT (Fig. 5).^251^ While targeting these signaling pathways has been an attractive strategy, unfortunately some phase I clinical trials showed that combined targeting of PI3K and MEK or mTOR signaling has little to no therapeutic window due to overwhelming toxicity prior to sufficient efficacy.^266,267^ Other mechanisms to inhibit c-Myc function in AML are being developed through suppression of c-Myc expression and activity.

#### Inhibition of c-Myc activity

Whether c-Myc promotes or represses the expression of a target gene depends on a complex network of co-factors. In order to translocate into the nucleus and bind to DNA, homotypic or heterotypic dimerization with its family members is required.^249,251^ c-Myc often dimerizes with Max (c-Myc family member) to bind to the promoter region of genes that it regulates (Fig. 5a). Development of small molecules that inhibit this dimerization have been challenging, but 10058-F4 was found to inhibit c-Myc through this mechanism and was demonstrated to increase sensitivity of AML cells to cytotoxic drugs, repress colony formation, promote differentiation, and induce apoptosis (Fig. 5a).^251,253^ Though promising in vitro, in vivo studies with this inhibitor failed due to poor PK and bioavailability.^257^ Fortunately, it has been a useful research tool for elucidating the vast control of c-Myc on different aspects of cancer, including AML.

HDAC inhibitors are potential novel therapies for the treatment of AML,^268^ which not only disrupt c-Myc activity but also suppress its expression (Fig. 5a).^269^ While dimerized with Max, c-Myc resides on the promoter of the pro-apoptotic gene *TRAIL* and represses its transcription by binding to Miz1 and Sp1 (Fig. 5a). HDAC inhibitors induce acetylation and displacement of c-Myc from the promoter and Sp1 is freed to promote transcription of *TRAIL* and induction of apoptosis in primary blasts derived from AML patients (Fig. 5a).^269^

#### Suppression of c-Myc expression

Overexpression of c-Myc in AML is rarely caused by mutations to the *MYC* gene itself. Therefore, a central theme in developing AML therapies that suppress c-Myc expression is by targeting its expression. Most cytokines, growth factors, and other mitogenic signals that utilize signaling cascades have been found to mediate c-Myc transcription. Several factors that have been tested as targets in AML and include the bromodomain and extraterminal (BET) domain-containing protein, Brd4, and other transcription factors.^269,270^ BET proteins are responsible for the preferential transcription of genes, including oncogenes such as *Bcl-2* and *MYC*.^271^ Since direct inhibition of c-MYC has proved difficult, this has led to the development of a class of small-molecule BET inhibitors, including JQ-1, which inhibits Brd4 from supporting c-Myc expression and has shown antileukemic activity against AML cells in in vivo models and has been used in preclinical studies (Fig. 5b).^256^ BET inhibitors have expanded and progressed due to their ability to be used in many different cancers and ones that have advanced to clinical trials in hematologic malignancies, including AML, are mivebresib (ABBV-075) (NCT02391480), FT-1101 (NCT02543879), birabresib (OTX015/MK-8628) in combination with azacitidine (NCT02303782), PLX51107 in combination with azacitidine (NCT02683395), INCB054329 (NCT02431260), RO6870810 (NCT02308761), and CPI0610 (NCT02157636, NCT01949883, NCT02158858).^271^ Mivebresib (ABBV-075), which targeted AML progenitor cells in vitro in combination with venetoclax,^272^ has completed a phase I trial in both advanced solid tumors and in AML and NHL. Published data on advanced solid tumors demonstrated a tolerable safety profile and induction of stable disease in some patients with malignant tumors, and early results from AML patients have also reported tolerability and antileukemic effects, though official analysis has yet to be published.^273,274^ Birabresib (MK-8628/OTX015), a first-in-class bromodomain inhibitor, was the first BET inhibitor to enter into phase I clinical trials where it was found to be well tolerated with a desirable toxicity profile and demonstrated some clinical activity in AML patients but failed to progress to phase II clinical trials.^275^ Similarly, INCB054329 failed clinical application during a phase I clinical trial in solid tumors and hematologic malignancies due to variability in PK. FT-1101 is an inhibitor of all four BET family members that is structurally unique from other BET inhibitors in the JQ-1 class.^276^ Its potency against leukemia cell lines accelerated it into a phase I clinical trial to assess safety, PK, pharmacodynamics, and clinical activity in patients with R/R AML or NHL. This trial is complete, and early published reports highlight acceptable safety as a monotherapy with modest clinical activity, though no data were given for the use in combination with azacitidine.^277^ CPI0610 is another BET inhibitor that demonstrated a BET-driven reduction in *MYC* gene expression and AML tumor growth inhibition in a xenograft study^278^ and is currently under evaluation in multiple phase I clinical trials in lymphoma and MM, and a phase II trial that is recruiting for acute leukemia, MDS/MPN, and myelofibrosis (NCT02157636, NCT01949883, NCT02158858). In a BRD4 profiling study, the compound PLX51107 was identified to be a unique BET inhibitor in its binding profile and demonstrated nanomolar potency against all four BET family members.^279^ Additionally, it was well tolerated in in vivo murine models, was much more potent than birabresib (MK-8628/OTX015), and has a much shorter half-life than birabresib (MK-8628/OTX015), which could lead to an improved therapeutic index as BET proteins have important roles in normal tissues and prolonged inhibition leads to negative side effects. This initial study found that PLX51107 significantly prolonged survival of mice in CLL mouse xenograft studies, highlighting potential for success in human studies. An additional study on PLX51107 investigated its use in MYC-driven lymphomas, namely, DLBCL, and found that PLX51107 drove both MYC downregulation in DLBCL cells and BIM-dependent apoptosis that promotes synergy with venetoclax (Bcl-2 inhibitor).^280^ Success of PLX51107 in preclinical studies have pushed it into a phase I clinical trial that is currently ongoing to evaluate safety, PK, pharmacodynamics, and clinical activity in combination with azacitidine AML and MDS patients. Though there is no preclinical data, a phase I trial on RO6870810, another BET inhibitor, was completed in 2017, though there is no official publication on the results of this trial and there is no phase II trial at this time. The development of this class of BET inhibitors has demonstrated how targeting the expression of c-Myc, rather than its activity, is a more practical strategy that has produced promising results thus far. Both preclinical and clinical studies have also found success in using a combinational approach of BET inhibitors with other approved AML therapies and the quickly developing landscape of these inhibitors offer unique opportunities for combination therapies that will be well tolerated clinically.

As mentioned previously, CDK9 is a key protein in P-TEFb and is responsible for promoting the expression of several oncogenes, including c-Myc, when it is recruited by Brd4 (Fig. 5a).^281^ Supporting this, voruciclib is a second-generation CDK9 inhibitor that has been demonstrated to downregulate c-Myc in AML cell lines and primary patient samples (Fig. 5b).^109^ Interestingly, it also synergistically enhances the antileukemic activity of venetoclax through this mechanism (confirmed by c-Myc inhibition with 10058-F4) and the combination prolonged survival in AML xenograft models. Voruciclib also downregulates Mcl-1, a common player in venetoclax resistance, though the downregulation of c-Myc proved to be more important in the enhancement of venetoclax activity than Mcl-1 downregulation.^109^ Voruciclib is currently being tested in a phase I, open-label, 3 + 3 dose escalation clinical trial for the treatment of AML and B cell malignancies post treatment with standard therapy (NCT03547115). As mentioned previously, AZD4573, a therapeutic being developed by AstraZeneca, is another CDK9 inhibitor in a phase I clinical trial to test safety, tolerability, and pharmacodynamics in hematologic malignancies (NCT03263637).^108^ AZD4573 is more cytotoxic in AML compared to solid tumors, and this could partially be due to its role in downregulating Mcl-1 expression. Whether this CDK9 inhibitor perturbs the expression of c-Myc in AML cells has yet to be confirmed. Even so, further clinical development of CDK9 inhibitors in AML is promising and offers an opportunity to affect multiple AML pathways in addition to CDKs, including Mcl-1 (apoptosis) and c-Myc. These inhibitors are especially hopeful when proposed in combination with standard chemotherapy or other targeted therapies.

Other oncogenic activators of c-Myc transcription are options for targeting. One of the most prominent of these oncogenic signaling cascades that leads to c-Myc expression is the PI3K/AKT pathway (Fig. 5b). A novel dual inhibitor of PI3K and HDAC, CUDC-907, almost completely abolishes c-Myc expression at low nanomolar concentrations in AML cells lines and primary patient samples in vitro as quickly as 4 h of exposure.^282^ CUDC-907 also significantly enhances the antileukemic activity of venetoclax (Bcl-2 inhibitor) both in vitro and in vivo by suppressing c-Myc expression and reducing Mcl-1 protein stability.^123^ Additional studies have demonstrated that inhibition of c-Myc plays a significant role in the molecular mechanism underlying synergy with venetoclax, possibly more so than downregulation of Mcl-1.^109^ Nevertheless, utilization of the parallel function of CUDC-907, HDAC inhibition, is an additional strategy to target c-Myc expression (Fig. 5). Analogous to CUDC-907, other HDAC inhibitors have been shown to decrease *c-Myc* transcripts in addition to inhibiting its function (Fig. 5b).^269^ This *c-Myc* transcript reduction may also be related to inhibition of HDAC6 as HDAC6 is known to support nuclear translocation of β-catenin that then binds other transcription factors to activate the *c-Myc* gene (Fig. 5a).^283,284^ Progressively, several HDAC inhibitors are under clinical investigation for the treatment of AML^268^ and promote another alternative method to c-Myc suppression.

Other strategies of c-Myc suppression involve manipulation of the nucleic acid structures within and surrounding the gene or mRNA transcript. ASOs suppress *c-Myc* expression by hybridizing to *c-Myc* mRNA and preventing translation (Fig. 5c).^251,285^ However, while delivery methods aided the protection of oligonucleotides from nucleases, specificity is often a challenge.^286^ A more practical approach to inhibiting transcription is by using small molecules to stabilize G-quadruplexes, which form near the promoter of *c-Myc* gene. A high guanine frequency in G-quadruplexes allows the formation of short-hair pin structures responsible for recruiting transcription factors to the site. The transcription factor NM23-H3/NDP kinase B is specifically essential for DNA cleavage into the single-stranded gene for transcription by RNA polymerase II.^287,288^ GQC-05 is a molecule that works to inhibit *c-Myc* transcription by stabilizing these G-quadruplexes and preventing splicing by NM23-H3/NDP (Fig. 5c). It has been reported to decrease *c-Myc* mRNA levels, increase DNA damage, and induce apoptosis in AML cell lines.^289^ However, this small molecule has only shown efficacy in vitro and has not been tested in vivo or with primary patient samples.

More progressive strategies to target c-Myc expression via regulation of c-Myc translation have been developed. This process is dependent on the recruitment of ribosomal subunits to its long 5’-untranslated region (UTR).^290^ Due to its long 5’-UTR*, c-Myc* mRNA translation requires the formation of the translation-initiating complex, eIF4F, which is phosphorylated through FLT3 activation of PI3K/AKT/mTORC1 signaling (Fig. 5b). Additionally, activation of mTORC1 leads to phosphorylation of S6RP, which enhances translation by recruiting 40S ribosome to *c-Myc* mRNA (Fig. 5c).^291^ Therefore, inhibitors that are able to inactivate the PI3K, AKT, or mTORC1 pathways would lead to the inhibition of *c-Myc* translation (Fig. 5c). To this notion, RTK inhibitors that suppress the activation of mTORC1 also repress the translation of c-Myc and have added mechanisms for targeting AML cells (Fig. 5c). This mechanism also highlights how the dual inhibitor of both PI3K and HDAC, CUDC-907, is able to potently suppress c-Myc expression (Fig. 5c).^282^

### DNA damage response (DDR) signaling network

DDR is the intricate system that maintains genomic integrity through regulation of the cell cycle and DNA damage repair. AML is characterized by high genomic instability, resulting in part by dysregulation of DDR-related genes. As such, AML cells have a targetable vulnerability. Thus investigation of therapies targeting the DDR have been under development.^292^

The cell cycle is regulated by checkpoints (CHKs) that can pause progression in order to repair DNA damage before entering the next phase. Ataxia-telangiectasia-mutated and ataxia-telangiectasia and Rad3 related (ATR) are activated by single- and double-stranded breaks, respectively, which then phosphorylate and activate CHK2 and CHK1, respectively. Activated CHK2 and CHK1 phosphorylates and inhibits CDC25 phosphatases, preventing CDC25 phosphatases from removing inhibitory phosphorylation of CDK1/CDK2 on Tyr-15, ultimately resulting in cell cycle arrest.^293–296^ As ATR and CHK1 inhibitors dysregulate cell cycle CHKs that are activated in response to DNA damage, preclinical studies demonstrate great promise for several ATR and CHK1 inhibitors in combination with DNA-damaging agents.^292,297–302^ There are currently ten active clinical trials (www.clinicaltrials.gov, accessed June 2020) for the CHK1 inhibitor prexasertib, including one phase I trial for refractory and recurrent AML (NCT02649764). The ATR inhibitors AZD6738, BAY1895344, berzosertib, M1774, and M4344 have a combined total of 46 active trials, though none for the treatment of AML. Unfortunately, despite promising preclinical data, ATR and CHK1 inhibitors have had little clinical success at treating AML. CHK1 inhibitors unfortunately cause compensatory activation of the ERK pathway, potentially interfering with expected antitumor activity.^302,303^

Another potential cell cycle CHK protein in which preclinical investigation has shown great promise is Wee1. Wee1 phosphorylates CDK1 and CDK2 on Tyr-15, preventing progression through G2/M and S phase.^304,305^ Additionally, Wee1 inhibition has been shown to induce DNA damage through the induction of replication stress.^306–315^ Similar to ATR and CHK1 inhibitors, Wee1 inhibitors have been used in combination with DNA-damaging agents and have shown preclinical promise.^292,314–316^ Additionally, Wee1 inhibition has been shown to synergize with CHK1 inhibition in AML cells.^298,309^ There are currently 17 active clinical trials for the Wee1 inhibitor adavosertib in solid tumors (www.clinicaltrials.gov, accessed June 2020). Unfortunately, a phase I trial of adavosertib in combination with belinostat for treating relapsed or refractory myeloid malignancies or untreated AML was terminated.

Thus far, selective inhibitors of ATR, CHK1, or Wee1 have yet to be approved for AML treatment. However, HDAC inhibitors have been shown to have anticancer activity through downregulation of CHK1, Wee1, BRCA1, and/or Rad51.^282,317–326^ There are currently several FDA-approved HDAC inhibitors, though none for the treatment of AML. Their benefit seems to require combination approaches.^327^ However, there are promising HDAC inhibitor combinations currently in clinical trial. There is a phase I trial of belinostat in combination with pevonedistat for the treatment of recurrent or refractory AML (NCT03772925) and a phase II trial for entinostat and azacitidine for AML patients who are aged ≥60 years and unfit for standard chemotherapy (NCT01305499). This trial is being conducted to determine whether sequential treatment will improve efficacy, as concurrent treatment did not.^328^ So far, it seems that pracinostat in combination with azacitidine may have the most promise. Based on encouraging phase I/II results,^1^ it is now undergoing phase III trial for adults with AML who are unfit for induction chemotherapy. While a direct inhibitor of DDR kinases has yet to show clinical promise for treatment of AML, targeting multiple aspects of the DDR using HDAC inhibitors in combination with azacytidine could potentially be added to the armamentarium of AML treatment.

### Targeting signaling pathways in LSCs

A major contributor to the incurable nature of AML is resistance to therapy often manifested as relapse from remission.^329^ Relapse can be cultivated from a small subpopulation of quiescent (G_0_ phase) LSCs that are derived from transformed hematopoietic stem/progenitor cells.^15,17,330–333^ Not only do these cells maintain the heterogeneity of this disease, but once a patient has achieved remission, surviving LSCs can differentiate and result in recurrence, following the cancer stem cell (CSC) theory. This theory states that CSCs are undifferentiated at the top of hierarchy and maintain the disease by generating the entire population of cancer cells.^333^ At the point of relapse, the LSC population expands and has significantly increased phenotypic diversity.^334^ LSCs were first identified in 1994 by Lapidot et al., who discovered that AML cells with the HSC phenotype, CD34^+^/CD38^−^, were the only cells that could generate AML in NOD/SCID mice.^335^ Over time, it was confirmed that this small subpopulation, with stemness cell surface antigen signatures, drive relapse in AML and are present at diagnoses.^333^ While LSCs are rare and heterogeneous, it is difficult to decipher molecular features of LSCs that are targetable. However, the fortitude of leukemia research has brought about hopeful strategies to reduce the incidence of relapse in AML and potentially improve the cure rate.

#### LSC metabolic homeostasis

**Fig. 6:**
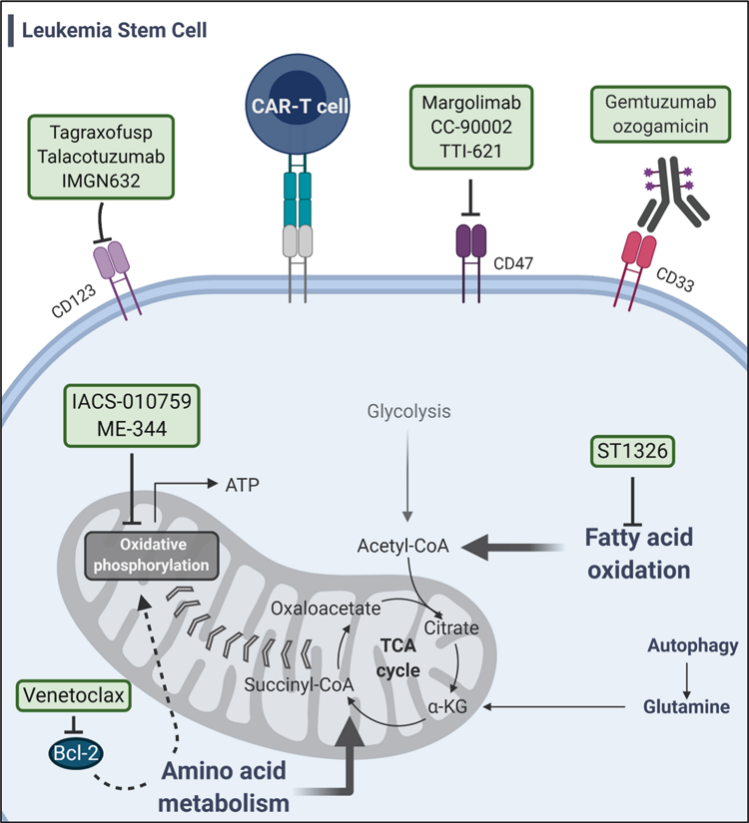
Targeting LSCs. LSCs have been found to express CD34, CD33, CD123, and CD47 surface antigens. Gemtuzumab ozogamicin is an antibody–drug conjugate that recognizes CD33 on LSCs and cellular internalization results in apoptosis via cytotoxic effects of the drug conjugate, ozogamicin. CAR-T cells that recognize surface antigens on LSCs are able to induce apoptosis in LSCs following binding. Tagraxofusp (IL-3 fusion toxin), talacotuzumab (anti-CD123 monoclonal antibody), and IMGN632 (CD123 antibody–drug conjugate) are CD123-specific immunotherapies. Magrolimab (anti-CD47 monoclonal antibody), CC-9002 (anti-CD47 monoclonal antibody), and TTI-621 (SIRPα-Fc fusion protein antibody) are CD47-specific immunotherapies. LSCs are uniquely reliant on oxidative phosphorylation for cell survival and amino acid metabolism and fatty acid oxidation are important for supplying intermediates for the TCA cycle in LSCs. Inhibition of mitochondrial respiration via direct ETC inhibitors (IACS-010759, ME-344) and indirectly via Bcl-2 inhibition (venetoclax) represents possible LSC therapeutics. ST1326 inhibits an enzyme in fatty acid oxidation (FAO) and limits FAO support of TCA cycle to indirectly inhibit mitochondrial respiration in LSCs

**Fig. 7:**
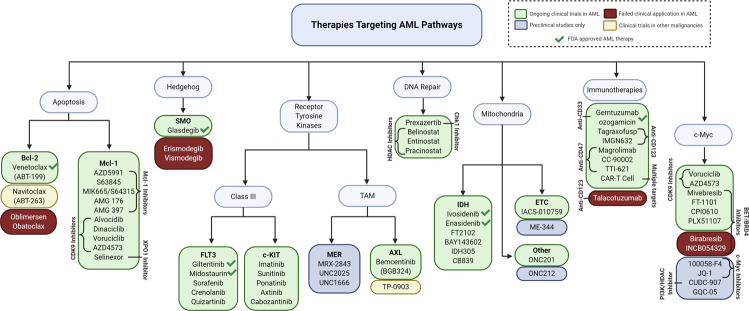
Therapies targeting AML pathways. Summary of newly approved therapeutics, therapeutics currently under clinical investigations, preclinical therapies, and failed clinical therapies in AML

To target LSCs, novel therapies must target a vulnerability of LSCs that is not shared with normal HSCs so that the risk of relapse can be reduced with minimal toxicity. Fortunately, a means to target LSCs is provided under the context of metabolic homeostasis. Simsek et al. previously revealed that LT-HSCs have low rates of mitochondrial respiration and HSCs can be isolated from BM aspirates based on this metabolic characteristic.^336^ Mitochondrial respiration relies on the ETC to carry out OXPHOS for the production of ATP,^337^ and in normal HSCs, inhibition of OXPHOS results in induction of glycolysis to maintain ATP production and cell survival. More recent studies have shown that normal HSCs rely on glycolysis when at peak self-renewal capacity.^336,338^ In contrast to normal HSCs, LSCs tend to depend on mitochondrial respiration (OXPHOS) for ATP production and have limited ability to fully utilize glycolysis when OXPHOS is inhibited (Fig. 6).^15,17,330^ While these undifferentiated cells do not proliferate as quickly as their bulk AML counterparts, ATP production is still essential for basic cellular functions and survival.^337^ Targeting OXPHOS has been shown to selectively target LSCs while sparing normal HSCs, and studies have found that LSCs rely on amino acid metabolism to support OXPHOS (Fig. 7).^15,17,186,330,336,339^

The inter-dependent relationship between the TCA cycle and the ETC is what makes OXPHOS possible (Fig. 6). In this relationship, the TCA cycle supplies the ETC with NADH and FADH2 and complex II (SDH) of the ETC is also an essential enzyme for the TCA cycle.^337,340^ In addition to normal glucose breakdown, amino acid and fatty acid metabolism supply intermediates for both TCA cycle and OXPHOS and are thus required for LSC survival (Fig. 6). This has made them a subject of interest to target LSCs while sparing normal HSCs.^330,332,337,339,340^ Since LSCs are not proliferating at a high rate like bulk AML cells, they utilize catabolic processes to derive metabolic intermediates via autophagy rather than anabolic reactions to generate the building blocks for a new cell.^331^ Jones et al. reported that LSCs with high concentrations of ROS (ROS-high blasts), which represent more matured blasts, are able to shift reliance to fatty acid metabolism under amino acid deprivation, but ROS-low LSCs, which represent more immature blasts, do not have this capability.^332^ They also found that relapsed LSCs are able to utilize fatty acid metabolism to escape amino acid loss and are more enriched for ROS-high LSCs.^332^ In fact, it is well documented that LSCs are supported by adipocytes in the BM microenvironment to supply lipids for fatty acid oxidation (FAO).^341^ Therefore, treating AML and eradicating LSCs early on during treatment is essential to avoid altered metabolic homeostasis in this small cell subpopulation. So far, elucidating the molecular mechanisms regulating metabolic homeostasis of LSCs has illuminated strategies for novel targeted therapeutics for the treatment of AML.

#### Targeting LSCs via metabolic regulation

Inhibition of amino acid metabolism suppresses the availability of intermediates for the TCA cycle and OXPHOS in LSCs (Fig. 6). Work by Jones et al. suggests that depletion of the availability of amino acids in the AML microenvironment selectively targets LSCs while sparing normal HSCs.^332^ More recently, this team has found that cysteine is particularly vital for LSC survival. Glutathione is exclusively derived from the metabolism of cysteine and supports glutathionylation of SDHA, an essential subunit of SDH/complex II.^340,342^ A more complete understanding of the LSC microenvironment and amino acid metabolic adaptation to stress has supported the development of agents that target this pathway. However, there is still the possibility of adaptation to amino acid depletion by shifting to FAO or metabolism (Fig. 6). This is found in ROS-low LSCs and those isolated from relapsed AML samples.^332,341^

LSCs and AML bulk cells that reside in the BM stimulate BM adipocytes to generate fatty acids through lipolysis. This promotes the salvage of these adipocyte-produced fatty acids by LSCs to generate intermediates for the TCA cycle and OXPHOS. BM-resident LSCs exhibit various characteristics that support this claim, such as expression of the fatty acid transporter CD36 and a pro-inflammatory phenotype. AML cells that are co-cultured with adipocytes also overexpress FABP4, a lipid chaperone, and knock down of this chaperone in AML cells prolonged survival in in vivo models.^343,344^ While studies regarding FAO and AML metabolic homeostasis are limited, it is well understood that FAO inhibition increases ROS production, induces apoptosis, and overcomes resistance to conventional AML chemotherapeutics via induction of the ISR mediator, ATF4.^341,345^ A key rate-limiting enzyme of FAO is carnitine *O*-palmitoyltransferase 1, which has several isoforms (CPT1A, CPT1B, and CPT1C). ST1326, a novel inhibitor of CPT1A, suppresses cell growth and induces mitochondrial damage and apoptosis in primary AML cells (Fig. 6).^346^ Several other inhibitors of FAO that work either directly or indirectly have been tested in other liquid cancers and solid tumors,^347^ but this strategy has yet to be confirmed against AML LSCs. Further studies are necessary to determine whether inhibition of FAO can eradicate AML LSCs and spare normal HSCs.

Other strategies include the inhibition of oncogenic signaling pathways, which have additional functions of regulating metabolism in AML and possibly LSCs. Complex I of the ETC, also known as NADH-ubiquinone oxidoreductase, is one of the two possible first steps of the OXPHOS pathway and supports a hypoxic cellular response. Conventional quinone-site inhibitors of complex I have been shown to be severely cytotoxic. These agents are particularly active in hypoxic conditions where normal mammalian cells can adapt by expressing hypoxia-inducible factors (HIFs).^348^ Since cancer cells use this same method to adapt to hypoxic tumor microenvironments, including leukemia cells in the BM, targeting HIFs has been a popular method of novel therapeutics. Through this pursuit, interestingly, the complex I inhibitor IACS-010759 was developed (mentioned in mitochondria pathway section above). Since its discovery, IACS-010759 has been demonstrated to target OXPHOS through inhibition of complex I (Fig. 6). This results in a reduction of the oxygen consumption rate of cells and induced apoptosis in AML xenograft models and primary blasts from R/R AML patients. A positive predictor is that IACS-010759 spared normal BM-derived mononuclear cells in in vitro studies, supporting a therapeutic window.^212^ The advancement of this novel complex I inhibitor has led to a phase I clinical trial for R/R AML (NCT02882321).

Since complex I deficiency has been reported to inhibit the release of cytochrome *c* and apoptosis-inducing factor, resistance to IACS-010759 may become a problem in AML.^349^ Fortunately, the selective Bcl-2 inhibitor, venetoclax, promotes cytochrome *c* release because of the tight regulation by this antiapoptotic protein. Recent literature has shown that inhibition of Bcl-2 targets LSCs through suppression of OXPHOS.^17,211,350^ The mechanism of OXPHOS suppression by venetoclax (Bcl-2 inhibitor) is unclear, but it is suggested that modification to amino acid metabolism might be the cause (Fig. 6). However, investigations into Bcl-2 and metabolic homeostasis is limited outside of the extensive work by Jones and colleagues.^17,332,351^ In several other cancers, Bcl-2 may act as an antioxidant in tumor cells by facilitating glutathione import to the mitochondrial matrix or directly reducing ROS generation.^351,352^ These findings support the earlier work of the same team (Lagadinou et al.), which found that overexpression of Bcl-2 protects ROS-low LSCs.^17^ Therefore, the combination of venetoclax and other OXPHOS-suppressing agents may be of interest, specifically for targeting this pathway in LSCs to reduce rates of relapse. Combination of ETC inhibitors with other approved therapies may also reduce the risk of toxicity that is a concern in this group of agents. Since Bcl-2 has also been shown to support OXPHOS in LSCs, we tested this combination in AML cell lines and primary patient samples. Our analysis determined that this combination is synergistic in AML cell lines in an OXPHOS-dependent manner.^353^ Therefore, this may also target LSCs since it overcomes a potential resistance mechanism to IACS-010759 and cross-links two methods of targeting OXPHOS.^17,351^

Inhibition of several other oncogenic signaling pathways is an interesting strategy to target both the bulk AML cell population and quiescent LSCs. PI3K/AKT and mTOR signaling have been extensively linked to supporting AML cell proliferation and survival, but they could also regulate metabolic homeostasis in AML cells and LSCs by several methods.^262^ Rapidly proliferating bulk AML cells rely on glycolysis for energy production, which is known to be supported through activation of mTORC1 by PI3K/AKT signaling.^347^ The PI3K/AKT signaling pathway is also one of the several mechanisms that activates c-Myc (in addition to MEK/ERK signaling). As a global transcription factor, c-Myc regulates a wide variety of cellular signaling and functional pathways. In terms of metabolism, c-Myc is known to upregulate glycolysis, but depending on the cell type and context it also promotes the expression of genes that support mitochondrial respiration. These enzymes include those that support fatty acid synthesis and glutaminolysis, such as fatty acid synthase and the glutamine transporter SLC1A5, as well as nuclear-encoded mitochondrial genes, promoting mitochondrial structure and biogenesis.^261,354,355^ However, literature that directly links c-Myc and OXPHOS lacks mechanistic studies and this link has yet to be tested in AML. Even if this connection is not established, targeting c-Myc may still help eradicate LSCs. Zhang et al. have provided some evidence that c-Myc, with the assistance of Sp1, modulates drug resistance in LSCs via promoting the expression of survivin.^356^ Therefore, c-Myc inhibition or depletion would be an intriguing tool for a regimen that targets both LSCs and the bulk AML cell population.

There are several potential regulators of PI3K/AKT signaling that may initiate chain reactions leading to metabolic shifts in LSCs. One is the microRNA, miR-126, which has been shown to control HSC quiescence and promote the self-renewal capabilities of LSCs in AML by targeting PI3K-AKT signaling.^357,358^ Interestingly, attenuation of miR-126 expression reduces survival of AML LSCs but does not affect normal HSCs, even though this microRNA supports HSC quiescence.^359^ Targeting miR-126 availability also reduced secondary engraftment of primary AML samples in xenograft models, further indicating that LSCs rely on the effects of miR-126.^360^ Since miR-126 suppresses the activity of PI3K-AKT signaling, this could be a major regulatory factor of cellular metabolism in AML. The knockdown of miRNA-126 may push LSCs toward glycolysis and differentiation into chemotherapy-sensitive AML cells, while sparing normal HSCs.

#### Immunotherapy

Over the past decade, immunotherapy has become a forefront in the novel strategies of cancer treatment. Surface marker targets are utilized in this form of therapy to promote activation of immune cell responses and numerous studies have been conducted in attempts to identify specific antigens on leukemic cells, mostly in CML. Despite this extensive work, only a few clinically relevant cell surface targets have been revealed to be uniquely expressed by AML LSCs. LSCs can generally be identified with the surface marker profile of CD34^+^/CD38^−^, but as normal HSCs can also exhibit this profile, CD34 is not an ideal target for therapy. Additionally, more recent literature has found that AML LSCs themselves are more heterogeneous than originally determined.^361^ Potential target antigens were difficult to decipher because they had to not only be selective for leukemic cells but they must also be homogeneously expressed on all CD34^+^ cells of leukemia samples regardless of CD38 expression. Additionally, some surface markers that are noted to be found on LSCs can also be found on various forms of normal myeloid progenitor cells and other normal cells, providing a small therapeutic window.^362^ Targeting AML based on surface markers often comes at a risk of myelosuppression, limiting the use in a clinical setting due to life-threatening adverse reactions, and this continues to be a concern with current AML immunotherapies. Ultimately cell surface antigens that have been identified include Siglec-3 (CD33), C-type lectin protein-1 (CLL-1), common leukocyte antigen (CD45), integrin associated-protein (IAP/CD47) and IL-3 receptor alpha-chain (IL-3RA/CD123).^362^ CD33 and CD123 are highly expressed in CD34^+^/CD38^−^ LSCs with small overlap in normal HSCs, making them promising targets.^361^

CD33 is believed to be found predominantly on leukemic blasts of AML patients and is expressed on most AML blasts, which makes it an attractive target for chimeric antigen receptor (CAR) T cell and mAb-based therapy (Fig. 6).^363^ In 1992, Bernstein et al. determined that CD33^−^ precursors isolated from AML patients who underwent long-term culture were non-clonal hematopoietic cells.^364^ Later, the findings of Bernstein et al. were confirmed when xenotransplantation studies showed that 99% of human AML cells that engrafted into the BM of NOD-SCID mice were CD33^+^.^365^ Eventually the use of gemtuzumab ozogamicin, a humanized anti-CD33 antibody, was found to bind to primary AML cells and was promising as an element for ADC-based therapies (Fig. 6). As mentioned previously, gemtuzumab ozogamicin is covalently bound to a cytotoxic drug that is able to induce DNA damage following internalization by the CD33 ligand. Gemtuzumab ozogamicin was originally approved for monotherapy in CD33+ AML patients aged ≥60 years in May 2000 but unfortunate features such as hepatotoxicity and BM toxicity caused the FDA to retract approval (Fig. 1).^363^ Further clinical trials were conducted to determine safety and efficacy of gemtuzumab ozogamicin at lower doses in combination with currently approved therapies, it was shown to prolong OS in AML patients, and it was well tolerated. This led to the approval of gemtuzumab ozogamicin in September 2017 for the treatment of newly diagnosed CD33-positive AML in adults and in pediatrics aged ≥2 years (Fig. 1).^61^ Currently gemtuzumab is under a phase 1 clinical trial in combination with venetoclax for R/R AML (NCT04070768).

CD123 (IL-3Rα) is an IL-3 receptor that promotes cell proliferation in response to IL-3 and is overexpressed in many hematologic malignancies, including AML.^366^ Its expression on normal hematopoietic cells has been characterized as low to absent and it has been identified as a unique marker on both on LSCs and AML bulk cells, making it an appropriate target for immunotherapies.^367^ CD123 expression is correlated with failure to achieve remission and worse overall prognosis^368,369^ and thus has been extensively studied as an immunotherapeutic target in AML. Further studies have identified overlap in overexpression of CD123 in patients who also have FLT3-ITD- and NPM1-mutated AMLs.^370^ The first CD123 targeting agent to reach FDA approval in December 2018 for the treatment of blastic plasmacytoid dendritic cell neoplasm (BPDCN), another myeloid malignancy with high expression of CD123, was tagraxofusp (SL-401). Tagraxofusp is a fusion toxin that is composed of a portion of the diptheria toxin fused to human IL-3. This breakthrough therapy approval was based on a multicenter, multicohort, open-label, single-arm clinical trial (NCT02113982) in patients with R/R BPDCN. This phase I/II clinical trial demonstrated a favorable toxicity profile for tagraxofusp and notably no myelosuppression was noted, reducing concerns for targeting normal hematopoietic cells and BM suppression. Tagraxofusp has also been supported in the treatment of AML as it had potent cytotoxicity against AML cells. Due to the relation of LSCs and minimal/measurable residual disease (MRD), tagraxofusp was evaluated in a phase I/II trial in patients with AML in first or second CR with MRD with the intent to lessen MRD and improve clinical outcomes (NCT02270463). Preliminary results from this trial were presented in December of 2017 and reported that in stage I of this study tagraxofusp had a safety profile that was similar to what was observed in other tagraxofusp clinical studies with no unexpected adverse events and targeting MRD with tagraxofusp has potential to improve outcomes in AML patients in remission with high risk of relapse.^371^ This trial was completed earlier this year. An upcoming trial with tagraxofusp in AML is a phase II trial in patients with CD123+ R/R AML that has not yet started recruiting (NCT04342962).

Other options for interfering with the CD123/IL-3 signaling pathways is using mAbs to CD123 that prevent the binding of IL-3 and are cytotoxic to CD123+ AML cells.^366^ CSL362 was thus developed to neutralize CD123 and displays antibody-dependent cytotoxicity against AML stem cells.^372^ This study also demonstrated that CSL362 had increased affinity and potency compared to CSL360, another CD123 antibody therapy that lacked clear antileukemic activity in a phase I study.^373^ Based on its success in AML xenograft studies,^372^ CLS362 entered into clinical studies and was renamed talacotuzumab. In recent reports on the clinical use of talacotuzumab, it demonstrated that as a monotherapy it has significant toxicity that led to a high rate of early treatment discontinuation and had an unfavorable risk/benefit profile.^374^ Similar conclusions were made in a phase II/III study comparing talacotuzumab in combination with decitabine vs decitabine alone. Only 10% of patients achieved CR on combination therapy compared to 11% of patients receiving decitabine alone, resulting in early termination of enrollment and discontinuation of treatment.^375^ Noted in these studies was the limitation of patients’ natural killer (NK) lymphocytes present and preclinical studies have suggested the use of human allogeneic NK cells in combination with talacotuzumab to promote NK cell antibody-dependent cell-mediated cytotoxicity.^376^

Another way to improve cytotoxicity of mAb therapies is using a CD123-targeting ADC. IMGN632 (ImmunoGen, Inc.) is an ADC composed of humanized anti-CD123 antibody linked to a cytotoxic compound (DNA mono-alkylating payload of the indolinobenzodiazepine pseudodimer).^377^ IMGN632 was found to have antileukemic effects without targeting myeloid progenitors and was cytotoxic against PDX models in ALL, which strongly supported its use as a clinical therapy. The combined use of IMGN632 and PARP inhibitors is synergistic in primary samples from R/R AML patients. A phase I/II trial is ongoing to assess safety, tolerability, PK, immunogenicity, and preliminary antileukemic activity of IMGN632 as a monotherapy in patients with CD123+ disease (AML, BPDCN, ALL) (NCT03386513). Preliminary updates on this monotherapy trial report that there is a manageable safety profile and broad therapeutic window in high-risk R/R AML and BPDCN patients, with no patterns of hepatotoxicity or cytopenias below the dose-limiting toxicities.^378^ In AML patients, 55% had a reduction in BM blasts and 20% achieved objective response (CR/CRi/MLFS).^378^ Most responders had failed prior intensive therapies and 62% had adverse-risk classification, and 23% were primary refractory. These encouraging results as a monotherapy promoted the development of a phase Ib/II clinical trial to determine the safety, tolerability, and antileukemic activity in combination with azacitidine and/or venetoclax in patients with relapsed and CD123+ AML (NCT04086264).

The development of a wide variety of approaches to target CD123 in AML has produced many promising results and has fueled the progression of numerous clinical trials. Combining this approach with other effective AML therapies offers an opportunity to eliminate both bulk AML cells and LSCs—potentially improving rates of long-term remission and survival in AML.

Another immunologic target identified in AML is CD47, which mainly functions as an anti-phagocytic signal that allows expressing cells to evade phagocytosis via macrophages and other phagocytes and has been found to be overexpressed in multiple hematologic malignancies, including AML.^379^ The expression of CD47 on AML cells promotes immune cell evasion and is related to poor prognosis. This understanding promoted the development of magrolimab (Forty Seven, Inc.), the first anti-CD47 antibody, which promoted both the innate and adaptive antitumor immune response in in vivo studies. CD47 has also been identified as an LSC marker, and anti-CD47 antibody immunotherapy reduced the burden of LSC progenitor cells in the BM of mice and prevented successful secondary engraftment of LSCs.^380^ Though CD47 is also expressed on many normal cells as well, toxicity is lower than expected because leukemic cells also express the necessary pro-phagocytic signals to promote immune responses, whereas normal cells typically do not. Red blood cells (RBCs), on the other hand, have been noted to be particularly sensitive to blocking CD47 as it regulates RBC clearance, and anemia is a concern with anti-CD47 therapies, though this adverse event is reported to be managed clinically with little complication.^381^ Currently, magrolimab is slated for five clinical trials, one in lymphoma, one in mycosis fungoides, one in MDS, and two in R/R AML. Of the two trials in AML, a phase I trial will determine the safety and tolerability of magrolimab as a monotherapy and in combination with azacitidine in R/R AML/MDS patients (NCT03248479) and the other is a phase Ib/II trial to study magrolimab in combination with azacitidine and venetoclax (NCT04435691). In 2019, preliminary results from this phase Ib trial were presented for the high-risk MDS (*n* = 18) and previously untreated AML (*n* = 25) patients with intermediate to poor risk. Magrolimab + azacitidine was well tolerated with a safety profile similar to what is expected with azacitidine, with some patients experiencing treatment-related anemia (37%), thrombocytopenia (26%), and neutropenia (26%).^382^ Though anemia was an expected adverse event, 69% of AML patients became RBC transfusion independent, demonstrating a tolerable toxicity profile for magrolimab.^382^ In AML patients evaluable for efficacy, 69% (11/16) had objective response with 50% achieving CR/CRi, 13% achieving a PR, and 31% with stable disease.^382^ Time to response was 1.9 months, which is more rapid than what is expected for azacitidine alone. Additionally, LSC frequency was evaluated via flow cytometry in the BM of these patients and LSCs were eliminated in 63% (10/16) of patients who had a clinical response.^382^ These results agree with preclinical data which demonstrate that magrolimab can target CD47+ LSCs in the BM. Response was favorable in TP53 mutant patients, highlighting efficacy in patients with poor prognosis.^382^ No median duration or OS has been reported yet. Since magrolimab, several other therapies designed to target CD47 or its macrophage receptor, SIRPα, have been developed and entered clinical trials.^381^ CC-90002, produced by Celgene, is another anti-CD47 antibody that was in a phase I clinical trial in R/R AML and high-risk MDS patients (NCT02641002) but was terminated due to it reportedly not offering a sufficiently encouraging profile for further dose escalation/expansion but is still in an open-label, phase I trial in patients with R/R solid and hematologic cancers in combination with rituximab, an anti-CD20 antibody immunotherapy (NCT02367196). TTI-621 (Trillium Therapeutics Inc.) is a SIRPα-Fc fusion protein antibody that blocks the CD47–SIRPα axis by binding to CD47 to enhance phagocytosis of tumor cells.^383^ Preclinical studies found that TTI-621 enhanced macrophage phagocytosis of both hematologic and solid tumor cells and spared normal cells and importantly only had minimal binding to RBCs—eliminating concern for anemia that is seen in anti-CD47 antibody therapies.^383^ It also demonstrated favorable results in both AML and lymphoma xenograft studies.^383^ An open-label, phase I trial of TTI-621 in patients with percutaneously accessible solid tumors or mycosis fungoides to evaluate safety profile of TTI-621 intratumoral injections was terminated (NCT02890368), but another phase I trial in R/R hematologic malignancies and selected solid tumors to investigate TTI-621 as a monotherapy and in combination with rituximab (anti-CD20 antibody) or nivolumab (anti-PD-1 antibody) is still currently recruiting (NCT02663518). Both preclinical studies and early results from clinical trials have demonstrated how targeting CD47, especially in combination with other approved AML therapies to promote phagocytosis, offers promising hope as an LSC-targeting therapy that could lead to durable remissions.

CAR-T cell therapies have also restored hope for CD33- and CD123-targeted therapies for AML (Fig. 6). These engineered T cells can recognize surface antigens in an antibody-specific manner and induce cell death upon activation.^384^ The first anti-CD19 CAR-T cell therapy (tisagenlecleucel) was FDA approved in 2017 for the treatment of relapsed pediatric B-ALL and brought a new form of immunotherapy options to leukemia. Similar to monoclonal-based therapies, CD33 and CD123 are ideal targets for CAR-T cell therapy because they are mostly AML cell- and LSC-specific antigens, though their expression on normal cells has limited therapy more than anti-CD19. Preclinical studies have found that targeting CD123 in addition to CD19 is able to more completely target LSC cells and prevent antigen loss relapse in in vivo studies.^385^ This further identifies the benefit of developing immunotherapies to target LSCs and the potential beneficial use that combination therapy can have for improved survival outcomes. The first reported clinical trial that demonstrated clinical activity of CAR-T cell therapy in AML was with CD28-ζ CAR directed to Lewis Y antigen.^386^ Though there was disease progression with this therapy, this study demonstrated that CAR-T cell therapy could be safely administered in AML patients and has pushed forward the development of other CAR therapies.

Currently there are 26 ongoing clinical trials to assess the use of CAR-T cell therapies in AML, including 12 that are specifically for anti-CD123 CAR-T, 5 for anti-CD33 CAR-T, and 3 that are investigating CAR-T cells that target multiple antigens (clinicaltrials.gov). Other targets include CLL-1, CD7, CD28, CD38, CD19, CD56, CD44v6, FLT3, NKG2D, and NKR-2. Early reports from an ongoing trial using CD28-ζ CAR-T cells targeting CD123 (NCT02159495) show that, of the two patients treated with dose level 1 (50M CAR+ T), one achieved a morphological leukemic-free state that lasted 2 months. Following this, the patient received a second infusion and had a blast reduction from 77.9 to 0.9%.^387^ Of the four patients on dose level 2 (200M CAR+ T) one patient achieved CR, one patient who had achieved CR prior to treatment maintained CR status, and the remaining two patients had reductions in blast count without achieving remission. Importantly, all toxicities were reversible and manageable, and there were no dose-limiting toxicities or related cytopenias.^387^ This first-in-human clinical trial of anti-CD123 CAR-T cell therapy demonstrated both clinical activity and safety of targeting CD123. Limitations of CAR-T cell therapy that are still being considered in AML are limiting CAR-T cell persistence following treatment to prevent long-term myelosuppression, immunosuppression induced by AML, and complications with CAR-T cell manufacturing.^388^ As more CAR-T cell clinical trials are underway, it will be interesting to assess both the continued safety of these therapies in patients as well as if this immunotherapy targets relapse-inducing LSCs and can possibly offer hope for improved patient survival.

## Concluding remarks

AML is an extremely heterogeneous disease and continues to have a poor prognosis, despite recent clinical advancements. Efforts have focused on identifying important signaling, metabolic, and homeostatic pathways that have shown potential for the development of antileukemic therapies. Here we have outlined the many ways in which the development of targeted therapies are progressing in the treatment of AML and are focused on diverse pathways that include: apoptosis, RTK signaling, hedgehog signaling, mitochondrial respiration and metabolism, DDR, transcriptional regulation, and immunotherapy targets. In addition, recent progression in the field has uncovered the important role that LSCs play in disease persistence and progression and potential avenues of targeting this population of AML cells to improve patient outcomes. Current clinical investigations highlighted here represent hope for new AML therapies to come (Fig. 7) and also demonstrate the importance of generating combinational therapies to better address the dynamic complexity of AML.
